# Supplementary material for: DNA Aptamers for the Characterization of Histological Structure of Lung Adenocarcinoma
Source: Mol Ther Nucleic Acids. 2016 Dec 31;6:150–62. doi: 10.1016/j.omtn.2016.12.004 (PMC5363495; doi:10.1016/j.omtn.2016.12.004)
Supplement: Document S1. Figures S1–S8 and Tables S1–S3 [file mmc1.pdf]

OMTN, Volume *6*

Supplemental Information

DNA Aptamers for the Characterization of Histological Structure of Lung Adenocarcinoma

Galina S. Zamay, Tatiana I. Ivanchenko, Tatiana N. Zamay, Valentina L. Grigorieva, Yury E. Glazyrin, Olga S. Kolovskaya, Irina V. Garanzha, Andrey A. Barinov, Alexey V. Krat, Gleb G. Mironov, Ana Gargaun, Dmitry V. Veprintsev, Sergey S. Bekuzarov, Andrey K. Kirichenko, Ruslan A. Zukov, Marina M. Petrova, Andrey A. Modestov, Maxim V. Berezovski, and Anna S. Zamay

Table S1. Sequences of aptamers. Where F: CTC CTC TGA CTG TAA CCA CG (the forward PCR primer) and cR:GCA TAG GTA GTC CAG AAG CC (the reverse-complement of the reverse PCR primer).

Aptamer	Sequence
LC-17	F-CTTTTGTCTTTAGCCGAATTTTACTAAGCCGGGCTGATCA-cR
LC-18	F-TGCCCGAACGCGAGTTGAGTTCCGAGAGCTCCGACTTCTT-cR
LC-224	F-CCGGTAAATTCTCCTGACGCCGGGGTAAGTTTCTGAAATG-cR
LC-2107	F-CGCGGTGAAGGGTATATCCACTGCGTCCCGTGCCGTCGGT-cR
LC-2108	F-CCCAGAGTCAGTGCGGCCCTTCCTTACAGTTTACCCCCGA-cR
LC-29	F-ATACCAAAGTCGTCTCCGCTCCGGTTGCACAACGAAGTTC-cR

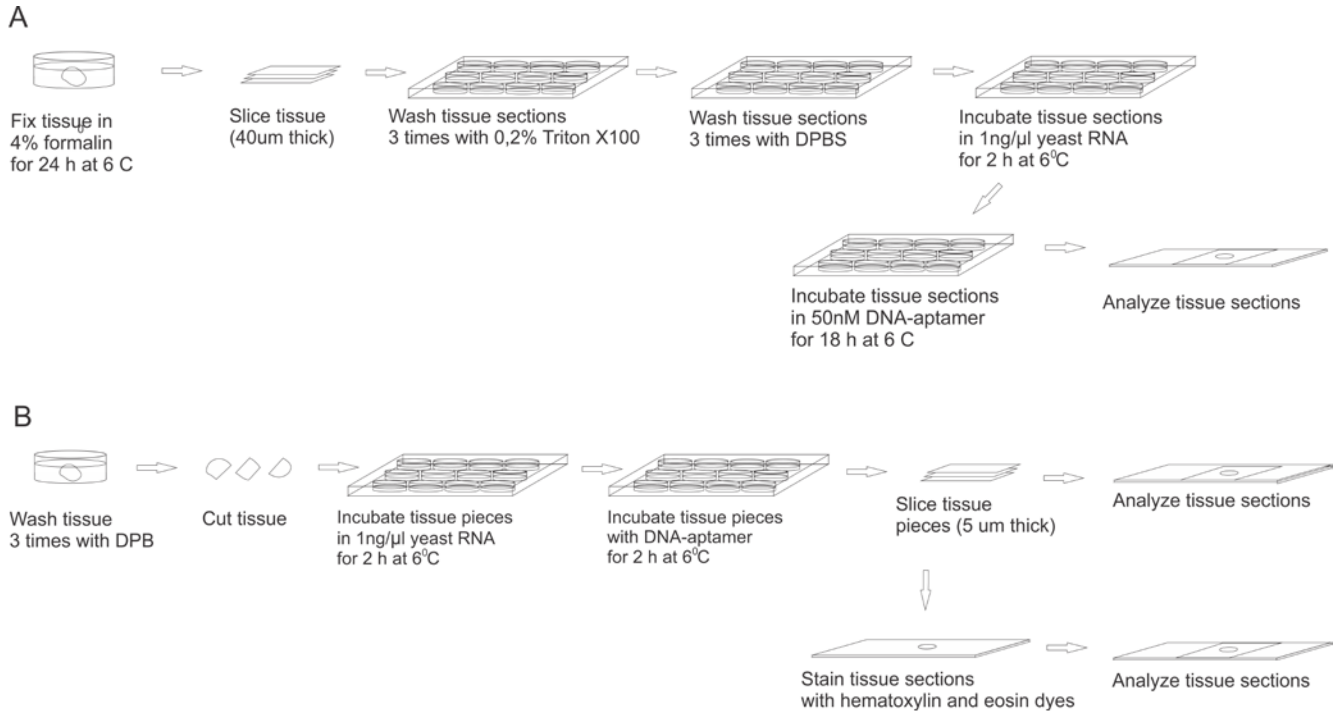

Figure S1. An overview of lung cancer tissue aptamer staining. (A) Tissue was fixed in 4% formalin, sliced, and washed 3 times with 0.2% Triton X-100 in DPBS. This was followed by incubation with 1 ng μL^{-1} yeast RNA in DPBS, incubation with 50 nM of a fluorescently labeled DNA-aptamer and analysis by microscopy. (B) Tissue was washed 3 times with DPBS and cut into smaller pieces. Next, the tissue pieces were incubated in 1 ngμL^{-1} yeast RNA in DPBS, and then incubated in sequence with DNA-aptamers. After washing, tissue pieces were sliced one of two adjacent slices were stained with hematoxylin and eosin dyes. All tissue sections were finally analyzed using fluorescent (or for better results laser scanning)and light microscopy.

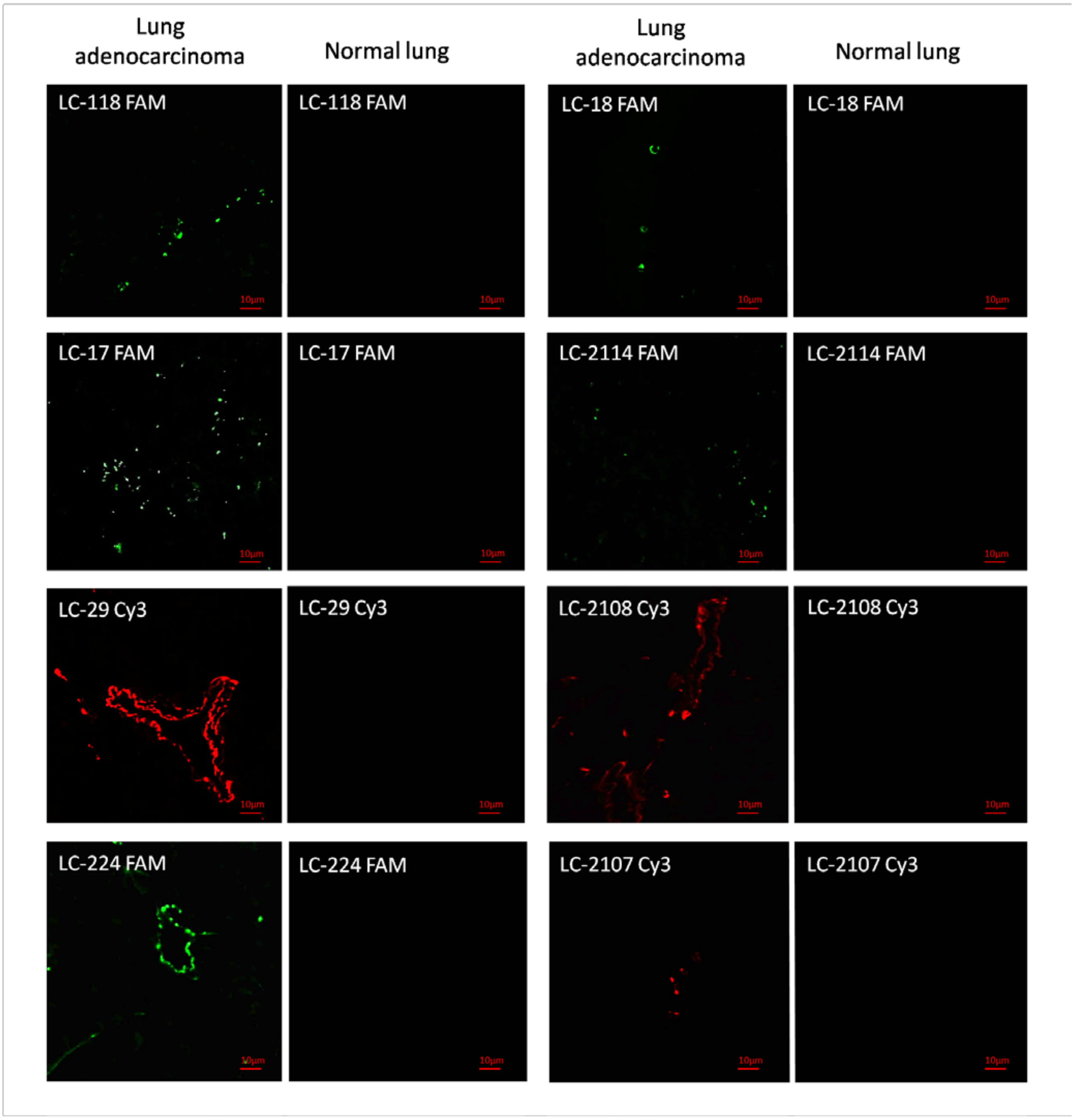

Figure S2. Cells (A) and blood vessels (C) alone and together (B) in lung adenocarcinoma tissue visualized with the following aptamers: LC-17 (A1, A2, B), LC-29 (C1, C3), LC-2108 (C2, C3). Laser scanning imaging (40X).

Table S2. Tissue structures in lung adenocarcinomatargeted by the aptamers.

	Aptamers to Lung Adenocarcinoma							
Targeted Structure	LC-17	LC-18	LC-29	LC-118	LC-224	LC-2107	LC-2108	LC-2114
Tumor Cells	+++	+++	+++	+++	--+	+++	---	+++
Connective Tissues	+++	---	---	---	+++	---	---	---
Blood Vessels	---	--+	---	---	+++	---	+++	---
Fibers	+++	---	---	---	+++	---	+++	---
Glandular Structures	---	+++	+++	---	---	---	---	---
Bronho-Alveolar Structures	---	---	+++	---	---	+++	---	---
Necrotic Tissues	---	+++	---	+++	---	---	---	---
Erythrocytes	---	---	---	---	+++	---	+++	---
Healthy LungCells	---	---	---	---	---	---	---	---
Healthy Connective Tissues	---	---	---	---	---	---	---	---
Cells in Tumor Margins	-+	++	+-	++	+-	++	-+	++

+ or - indicate binding of the aptamer to correspondent structure element in lung adenocarcinoma tissues derived from one patient.

Table S3. Pairwise alignment of lamin-A/C (P02545) and vimentin (P08670) protein sequence performed using program Protein Blast.

Range 1: 99 to 416 GenPept Graphics ▼ Next Match ▲ Previous Match

Score	Expect	Method	Identities	Positives	Gaps
131 bits(330)	4e-37	Compositional matrix adjust.	107/367(29%)	194/367(52%)	49/367(13%)

```
Query  27   TRLQEKEDLQELNDRLAVYIDRVRSLETENAGLRLRITESEEVVSREVSGIKAAYEAELG  86
            TR  EK +LQELNDR A YID+VR LE +N   ++ + E E++  +  S +   YE E+
Sbjct  99   TRTNEKVELQELNDRFANYIDKVRFLEQQN---KILLAELEQLKGQGKSRLGDLYEEEMR  155

Query  87   DARKTLDSVAKERARLQLELSKVREEFKELKARNTKKEGDLIAAQARLKDLEALLNSKEA  146
            + R+ +D +  ++AR+++E   + E+   L                R K  E +L  +EA
Sbjct  156  ELRRQVDQLTNDKARVEVERDNLAEDIMRL----------------REKLQEEMLQREEA  199

Query  147  ALSTALSEKRTLEGELHDLRGQVAKLEAALGEAKKQLQDEMLRRVDAENRLQTMKEELDF  206
                                      E  L   ++ + +  L R+D E ++++++EE+ F
Sbjct  200  --------------------------ENTLQSFRQDVDNASLARLDLERKVESLQEEIAF  233

Query  207  QKNIYSEELRETKRRHETRLVEIDNGKQREFESRLADALQELRAQHEDQVEQYKKELEKT  266
             K ++ EE++E + + + + V+ID    +     L  AL+++R Q+E    +  +E E+
Sbjct  234  LKKLHEEEIQELQAQIQEQHVQIDVDVSK---PDLTAALRDVRQQYESVAAKNLQEAEEW  290

Query  267  YSAKLDNARQSAERNSNLVGAAHEELQQSRIRIDSLSAQLSQLQKQLAAKEAKLRDLEDS  326
            Y +K  +  ++A RN++ +  A +E  + R ++ SL+  ++ L+    + E ++R++E++
Sbjct  291  YKSKFADLSEAANRNNDALRQAKQESTEYRRQVQSLTCEVDALKGTNESLERQMREMEEN  350

Query  327  LARERDTSRRLLAEKEREMAEMRARMQQQLDEYQELLDIKLALDMEIHAYRKLLEGEEER  386
             A E    +  +   + E+  M+  M + L EYQ+LL++K+ALD+EI  YRKLLEGEE R
Sbjct  351  FAVEAANYQDTIGRLQDEIQNMKEEMARHLREYQDLLNVKMALDIEIATYRKLLEGEESR  410

Query  387  LRLSPSP  393
            + L P P
Sbjct  411  ISL-PLP  416
```

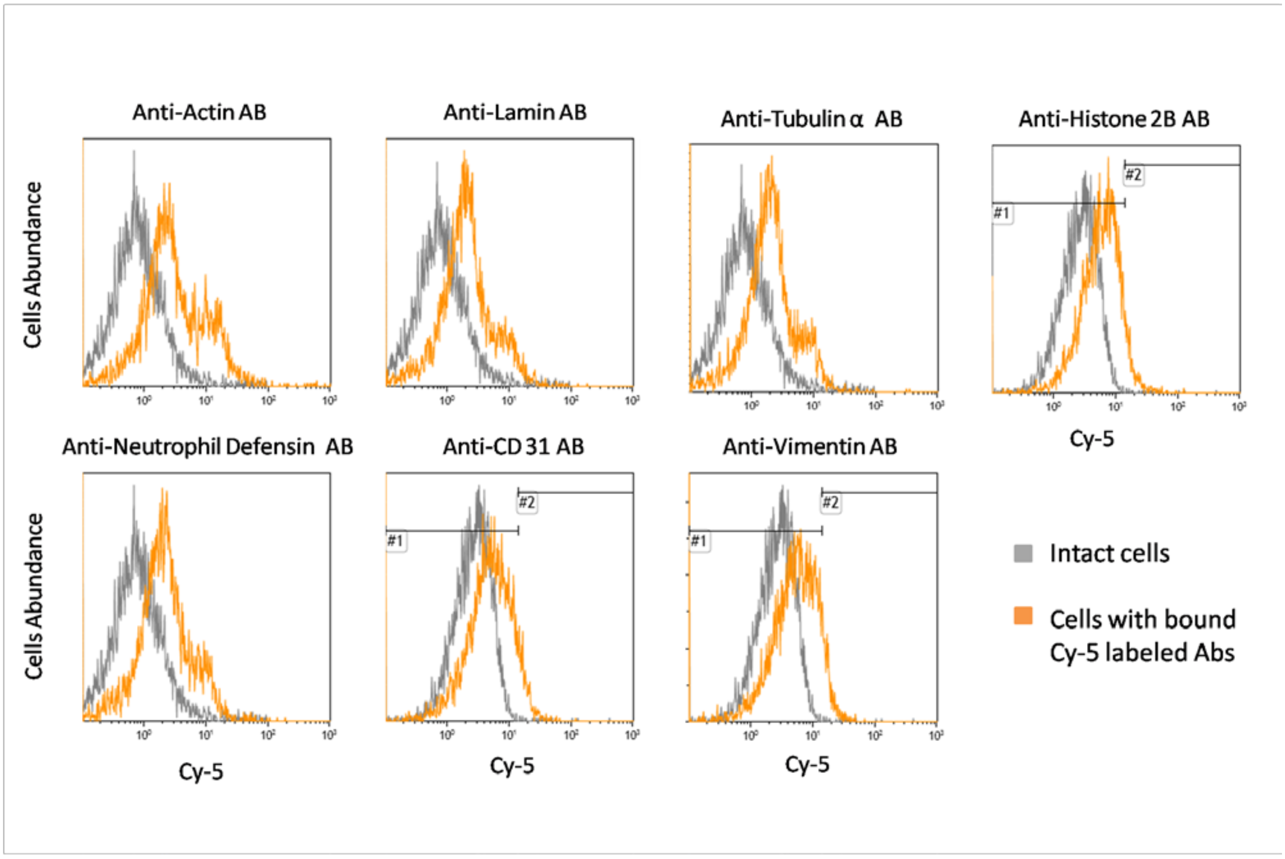

Figure S3. Flow cytometry histograms indicating binding of different antibodies (which have been used for aptamer replacement analyses) to lung adenocarcinoma cells. Gray curve corresponds to intact lung adenocarcinoma cells, orange - cells bound with Cy-5 labeled antibodies.

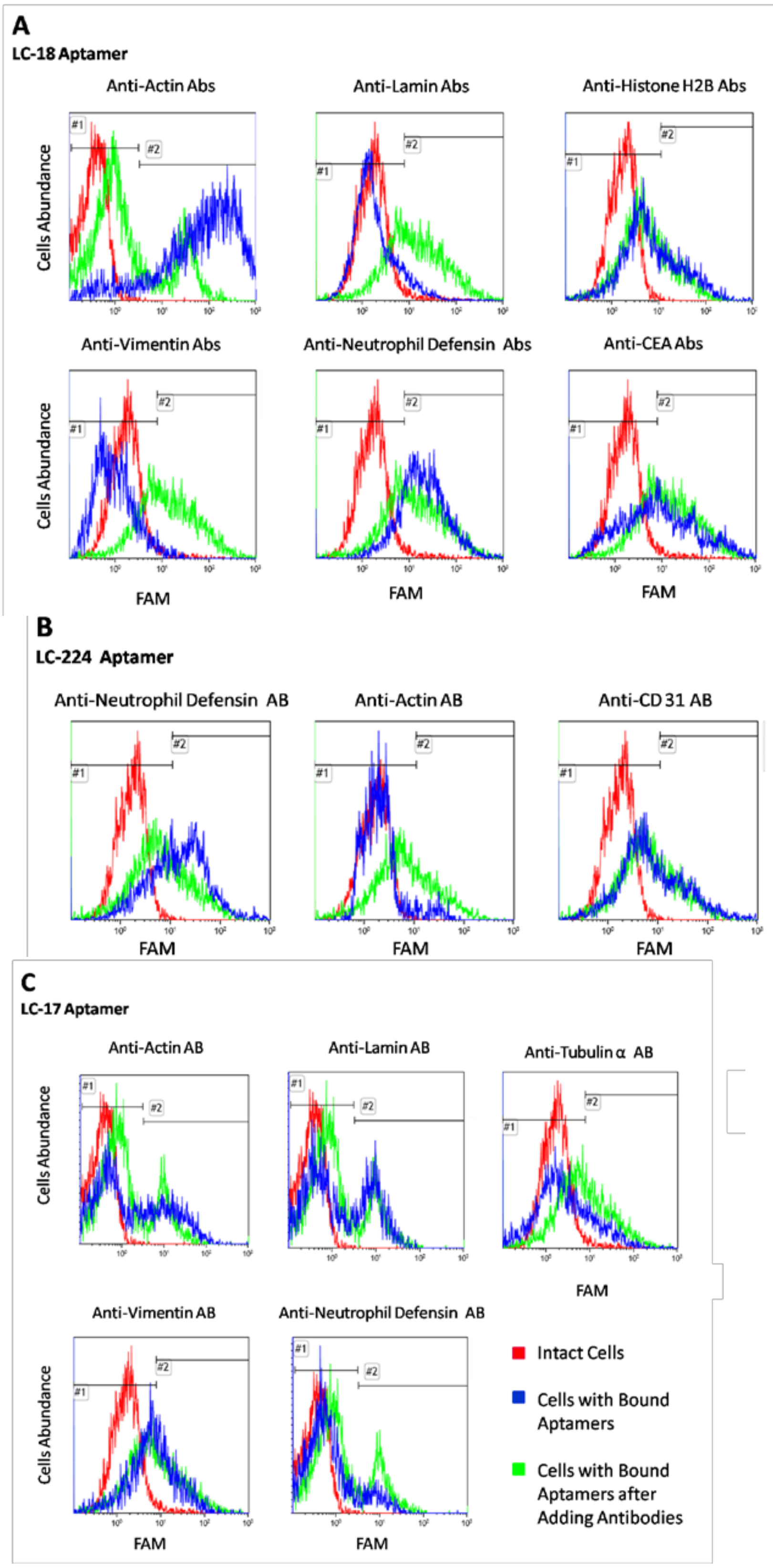

Figure S4. Flow cytometry histograms indicating binding of the aptamers LC-18 (A), LC-17 (B), LC-224 (C) to lung adenocarcinoma cells and its replacement with different antibodies (to correspondent proteins which were identified as aptamer-associated targets). Blue curve corresponds to intact lung adenocarcinoma cells, green to cells bound with the aptamer LC-18 (50nM) and blue - the same sample after incubation with 2 ng μL^{-1}Cy-5 labeled antibodies for 30 min with shakingat 37°C.

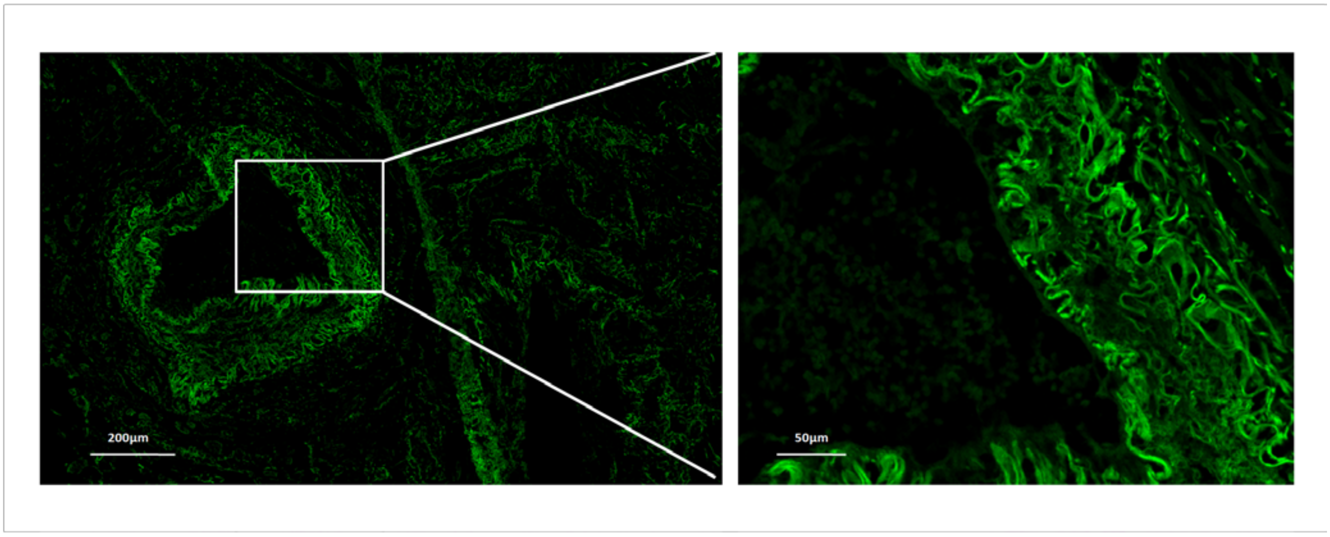

Figure S5. Binding of aptamer LC-224 to arteries and connective tissues: elastic, reticular and collagen fibers from patients with lung adenocarcinoma. Scanning the section (20X).

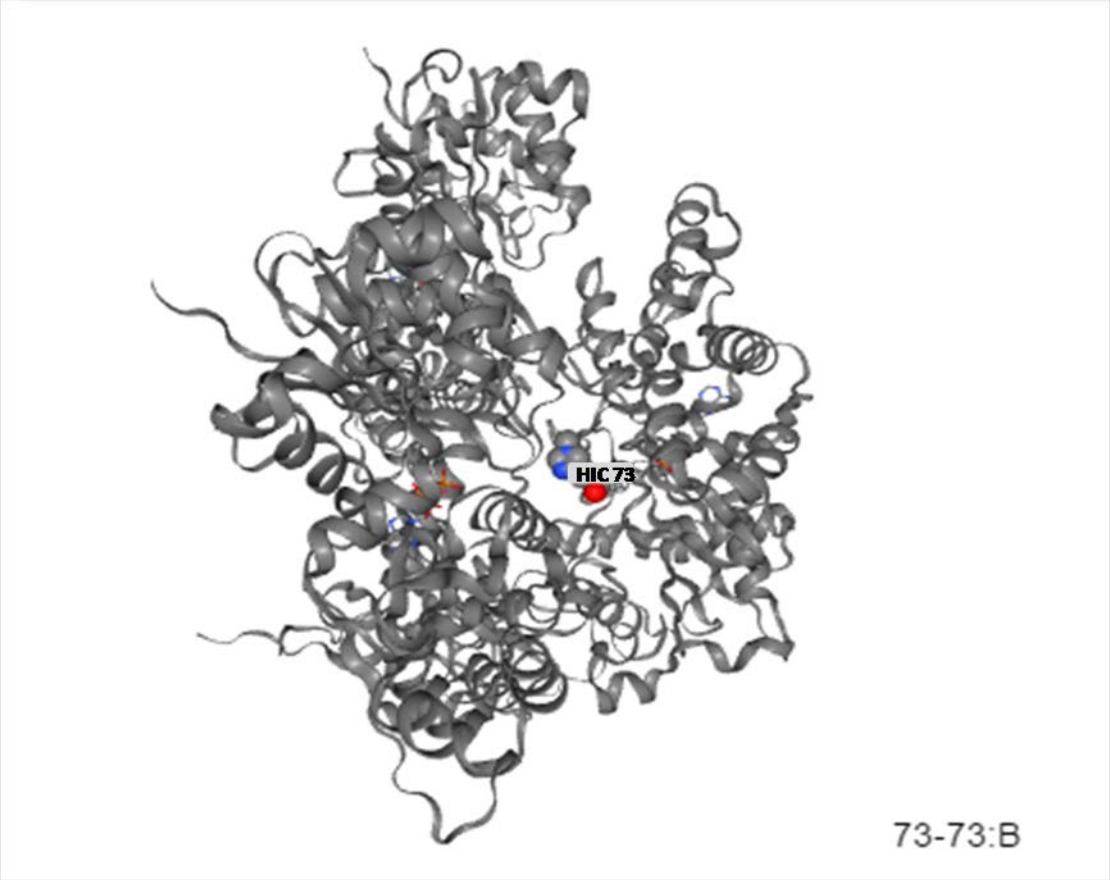

Figure S6. Methylation of 73 histidine in actin, cytoplasmic 1, protein feature view of PDB entries mapped to a UniProtKB sequence. Source:www.rcsb.org/pdb/protein/P60709

A

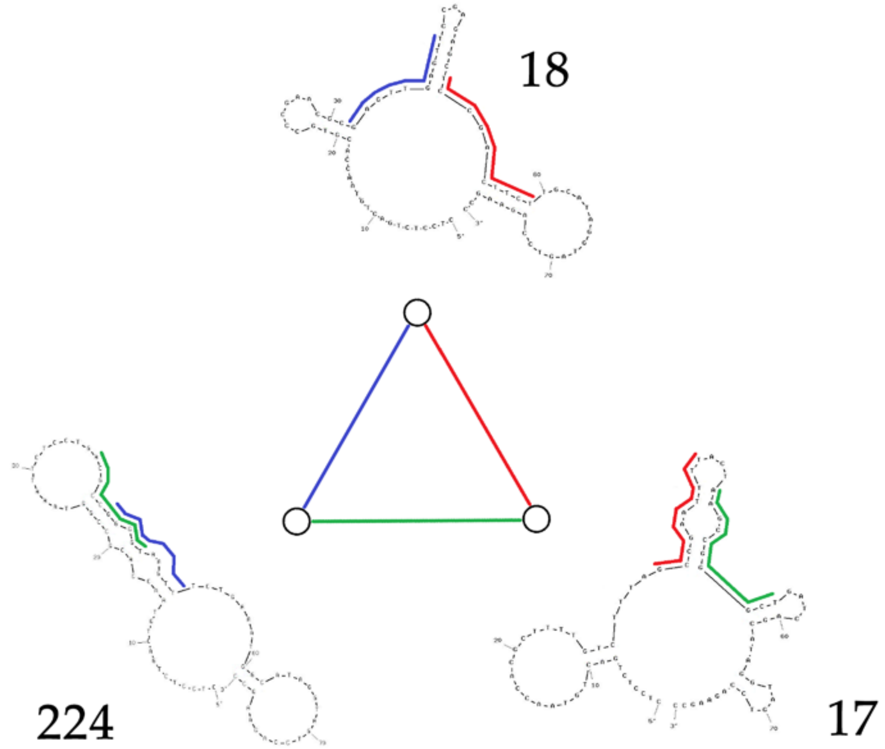

B

17 CTCCTCTGACTGTAACCACGCTTTTGTCTTTAGCCGAATTTTACTAAGCCGGGCTGATCAGCATAGGTAGTCCAGAAGCC

18 CTCCTCTGACTGTAACCACGTGCCCGAACGCGAGTTGAGTTCCGAGAGCTCCGACTTCTTGCATAGGTAGTCCAGAAGCC

224 CTCCTCTGACTGTAACCACGCCGGTAAATTCTCCTGACGCCGGGGTAAGTTTCTGAAATGGCATAGGTAGTCCAGAAGCC

Figure S7. Similar regions for each pair of the aptamers. 10-nucleotide regionsthat differ in the smallest possible number of symbols (from 3 to 2 difference) are shown in primary structure (A) and in the intended secondary structure (B). Each color corresponds to a similar region in a pair: LC-17 and LC-18 – red line; LC-17 and LC-224 – green line; LC-224 and LC-18 – dark blue line.The three aptamers were compared with each other in pairs and we observed thataptamerpairs have similar 10-nucleotide regions. These are shown in primary aptamer structures (B) and in the intended secondary structures (A). Each aptamer has a similar region with other aptamers; aptamer LC-17 has similar regions with LC-18 and LC-224. Despite some similarities in the primary structures, the secondary structures of closely related regions are different.

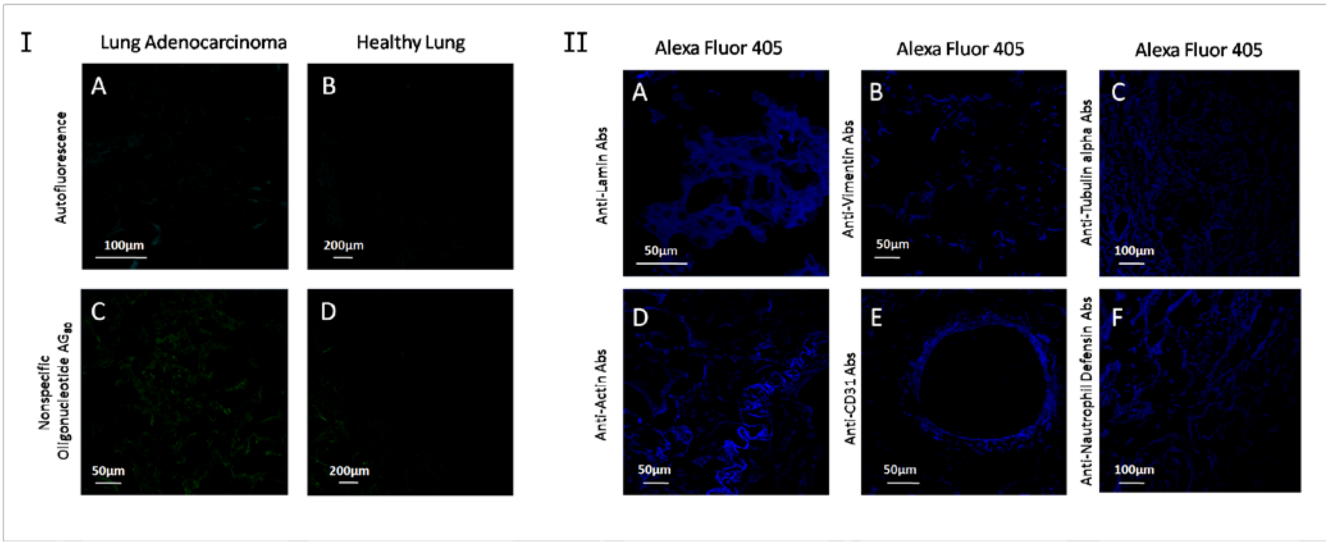

Figure S8. Negative (I) and positive (II) controls for aptahistochemical staining.(I)Autofluorescence of the tissuesections of lung adenocarcinoma (A) and healthy lung (B); binding of non-specific oligonucleotide $(AG)_{40}$to (A) lung adenocarcinoma, (B) healthy lungtissuesections. (II) Positive controls staining of lung adenocarcinoma with antibodies: anti-lamin (A), anti-vimentin (B), anti-tubulin alpha (C), anti-actin (D), anti-CD31 (E), anti-neutrophil defensin (F).
