# Supplementary material for: DNA Aptamers for the Characterization of Histological Structure of Lung Adenocarcinoma
Source: Mol Ther Nucleic Acids. 2016 Dec 31;6:150–62. doi: 10.1016/j.omtn.2016.12.004 (PMC5363495; doi:10.1016/j.omtn.2016.12.004)
Supplement: Document S2. Article plus Supplemental Information [file mmc2.pdf]

Molecular Therapy
Nucleic Acids
Original Article

DNA Aptamers for the Characterization of Histological Structure of Lung Adenocarcinoma

Galina S. Zamay,[1,2] Tatiana I. Ivanchenko,[1] Tatiana N. Zamay,[1] Valentina L. Grigorieva,[1] Yury E. Glazyrin,[1] Olga S. Kolovskaya,[1,2] Irina V. Garanzha,[1,2] Andrey A. Barinov,[6] Alexey V. Krat,[4] Gleb G. Mironov,[5] Ana Gargaun,[5] Dmitry V. Veprintsev,[1,2] Sergey S. Bekuzarov,[3] Andrey K. Kirichenko,[1] Ruslan A. Zukov,[1,4] Marina M. Petrova,[1] Andrey A. Modestov,[1,4] Maxim V. Berezovski,[5] and Anna S. Zamay[1,2]

[1]Laboratory for Biomolecular and Medical Technologies, Krasnoyarsk State Medical University, Krasnoyarsk 660022, Russia; [2]Institute of Chemistry and Chemical Technology of the Siberian Branch of the Russian Academy of Science, Krasnoyarsk 660036, Russia; [3]Krasnoyarsk Regional Clinical Pathological Anatomical Bureau, Krasnoyarsk 660022, Russia; [4]Krasnoyarsk Regional Clinical Cancer Center, Krasnoyarsk 660022, Russia; [5]Department of Chemistry and Biomolecular Sciences, University of Ottawa, Ottawa, ON K1N 6N5, Canada; [6]OPTEC Group, Novosibirsk 630090, Russia

Nucleic acid aptamers are becoming popular as molecular probes for identification and imaging pathology and, at the same time, as a convenient platform for targeted therapy. Recent studies have shown that aptamers may be effectively used for tumor characterization and as commercially available monoclonal antibodies. Here we present three DNA aptamers binding to whole transformed lung cancer tissues, including tumor cells, connective tissues, and blood vessels. Protein targets have been revealed using affinity purification followed by mass spectrometry analyses, and they have been validated using a panel of correspondent antibodies and 3D imaging of tumor tissues. Each of the proteins targeted by the aptamers is involved in cancer progression and most of them are crucial for lung adenocarcinoma. We propose the use of these aptamers in aptahistochemistry for the characterization of the histological structure of lung adenocarcinoma. The value of the presented aptamers is their application together or separately for indicating the spread of neoplastic transformation, for complex differential diagnostics, and for targeted therapy of the tumor itself as well as all transformed structures of the adjacent tissues. Moreover, it has been demonstrated that these aptamers could be used for intraoperative tumor visualization and margin assessment.

INTRODUCTION

Aptamers are becoming well admired for diagnostic and therapeutic applications. Recent studies have shown that aptamers might be effectively used for tumor characterization and as commercially available monoclonal antibodies.[1–3] Immunohistochemistry is one of the most commonly applied clinical methods for tumor diagnosis and differentiation. Among other histological methods, immunostaining of fixed tissue sections is the most popular for the detection of cancer-related biomarkers.[1]

Recent advances in targeted therapy of lung adenocarcinoma with selective tyrosine kinase inhibitors, selective inhibitors of epidermal growth factor, and receptor kinase activity of anaplastic lymphoma sufficiently improved the results of the treatment. The hallmarks of neoplastic disease include increased proliferation, evasion of growth suppressors, cell death resistance, activation of replicative immortality, angiogenesis, and activation of invasion and metastasis.[4] Due to the numerous pathologies in cancer tissue, histological detection of specific biomarkers is almost indispensable for diagnosis confirmation of malignancy.[1] In immunohistochemistry, a panel of different antibodies, such as carcinoembryonic antigen (CEA), gastrin-releasing peptide (GRP), cytokeratin (CK) 5/6, thyroid transcription factor 1 (TTF-1), p63, and thrombomodulin (CD141), is applied for biomarker detection on tumor cells.[5,6]

Cellular metabolism, mutational events, and multiple signaling pathways are crucial in understanding cancer.[7] Therefore, the detection of biomarkers on cancer cells is one of the main indicators of a neoplastic process, which may be facilitated by using antibodies and/or aptamers. Histological characterization of tumor vessels is also important, because angiogenesis is one of the key processes in tumor growth and progression.[8] Normalizing abnormal growth of tumor vessels inhibits tumor and metastasis.[8,9]

Aptamers may be effectively used as molecular probes for staining histological tissue sections and biomarker identification of tumor cells and abnormal blood vessels. At the present time, the prospects of aptamer application for the detection of cancer cells and proteins have been shown multiple times.[2,10–14] Often, tumor biomarkers are identified on surfaces of free-floating cells with the help of flow cytometry[15–18] and fluorescent and confocal microscopy.[2,3,19] However,

Received 4 April 2016; accepted 5 December 2016;
http://dx.doi.org/10.1016/j.omtn.2016.12.004.

Correspondence: Anna S. Zamay, Laboratory for Biomolecular and Medical Technologies, Krasnoyarsk State Medical University, 1 P. Zheleznyaka str., Krasnoyarsk 660022, Russia.
E-mail: aszamay@gmail.com
Correspondence: Maxim V. Berezovski, Department of Chemistry and Biomolecular Sciences, University of Ottawa, Ottawa, ON K1N 6N5, Canada.
E-mail: maxim.berezovski@uottawa.ca

This is an open access article under the CC BY license (http://creativecommons.org/licenses/by/4.0/).

aptamer applications for histological tissue section staining are still rarely described. Recently, several tumor tissue section staining methods have been reported with the following aptamers: aptamer SYL3C specific to the epithelial cell adhesion molecule (EpCAM) antigen,[3] an aptamer to CD30 expressed by lymphoma cells,[1] aptamer TTA1 to tenasin-C extracellular matrix protein,[8] and aptamer AX102 to the platelet-derived growth factor B (PDGF-B) that plays an important role in angiogenesis.[9]

Our current research describes the application of synthetic DNA aptamers, selected prior to postoperative lung cancer tissue,[20] for histochemical analyses of lung cancer tissues and visualization of tumor cells, abnormal blood vessels, and connective tissues. The aptamers bind to lung cancer cells as well as elastic fibers, which constitute a major part of the blood vessel walls. We found the protein targets of these aptamers using mass spectrometry and validated using a panel of correspondent antibodies. Furthermore, we describe a method for simultaneously staining tumor cells and blood vessels in lung cancer tissue, as well as protocols for formalin-fixed, no paraffin-embedded tissue staining with a single aptamer as an imaging probe and for simultaneous staining of frozen tissues with three different DNA aptamers. Moreover, it has been demonstrated that these aptamers could be used for intraoperative tumor visualization.

RESULTS

Previously, we selected DNA aptamers to postoperative whole minced human lung adenocarcinoma tissues, including tumor cells, blood cells, blood vessels, and connective tissues. Four aptamer clones were found to bind to lung adenocarcinoma cells and circulating tumor cells derived from patient's blood.[20] In this study, our scope was to select for aptamers capable of binding to other tumor structures, such as abnormal blood vessels and transformed connective tissues. We proposed the use of these aptamers in immunohistochemistry-like assays (aptahistochemistry) for tumor tissue characterization as well as for tumor intraoperative visualization and targeted lung adenocarcinoma therapy. Aptamer staining was carried out according to the scheme depicted in Figure S1.

First, we verified the selectivity of different aptamers (corresponding DNA sequences are presented in Table S1) to lung adenocarcinoma tissue structures by staining formalin-fixed tissue sections. Aptamer LC-18 with K_D = 38 nM was a good representative for labeling cancer cells, aptamer LC-224 (K_D = 96 nM) bound to transformed connective tissues and blood vessels in tumors, and LC-17 (K_D = 14 nM) bound to both connective tissue fibers and cells. Most importantly, none of the presented DNA aptamers bound to structure elements and cells in normal lung tissue (Figure S2; Table S2).

The interaction among tumor cells, connective tissues, extracellular matrix, chemokines, and blood vessels plays a major role in cancer progression.[21] Three aptamers described in this research target the most important tumor structures, such as connective tissues and fibers as well as blood vessels; therefore, they could be utilized for the characterization of complex tumors, cancer research, and/or targeted therapy (Table S2). Some aptamers simultaneously bind to different tumor elements; this may be because the oligonucleotides bind to the same protein expressed by different cells or one aptamer has several protein targets.

In our previous work, we identified aptamer-associated biomarkers for aptamers LC-17 and LC-18; these biomarkers were purified from adenocarcinoma cells derived from three patients.[20] Here we used whole, minced, and lysed lung adenocarcinoma tissues to isolate the proteins associated with these aptamers in order to keep all possible protein targets on connective tissues and blood vessels. Moreover, we increased the number of patients with adenocarcinoma to increase the statistical significance of the results in these experiments. The updated list of the aptamer-associated proteins for LC-17, LC-18, and LC-224 is presented in Table 1. LC-18, for which the tumor-associated biomarkers (identified for the tumor cells) are histone H2B and neutrophil defensin 1 and 3, binds to cancer cells and circulating tumor cells (CTCs).[20] The additional proteins in the list of proteins associated with this aptamer were discovered using minced tumor tissues (Table 1).

Similarly, for aptamer LC-17, more proteins were uncovered as possible aptamer-associated targets. We noticed that several proteins, such as actin, cytoplasmicneutrophil defensin 1, and heat shock protein beta-1, are common targets for all aptamers in our experiments, which may be due to non-specific binding. It was not surprising that all identified proteins have been found in tumor tissues and play various roles in cancer progression.

Aptamer target validation was performed using flow cytometry by displacing the aptamers for cancer cells with the correspondent antibodies (Table 1; Figure S4). Incubation with each antibody alone showed good binding with minced tissues (Figure S3). These experiments were performed at 37°C to ensure cellular internalization of the aptamers and antibodies. Additionally, aptamer-specific binding was validated by co-staining lung adenocarcinoma sections with aptamers and antibodies, which bind to the relevant aptamer-associated proteins found by mass spectrometric analyses (Figures 1, 2, and 3). These experiments were done using tissues from the third and fourth patients.

High concentrations of anti-lamin antibodies completely replaced aptamer LC-18 bound to adenocarcinoma cells derived from one patient (Figure 1, IIA) and partially (73% and 56%) displaced in two other patients (Table 1). Vimentin also was verified to be the target for LC-18 for two of three patients (Table 1), as anti-vimentin antibodies displaced most of the aptamer. Antibodies against other proteins identified as aptamer-associated proteins using mass spectrometric analyses did not displace LC-18 from adenocarcinoma cells (Figure S4A).

Microscopic analyses revealed co-localization of aptamer LC-18 with anti-vimentin antibodies in tumor cells outside and inside the blood vessels (yellow arrows in Figure 1, IB4) and elastic fibers (blue arrow

Table 1. Protein Biomarkers Associated with Aptamers to Lung Adenocarcinoma

Aptamer LC-17 K_D = 14 nM Tumor Cells, Connective Tissues F-CTTTTGTCTTTAGCCGAATTT TACTAAGCCGGGCTGATCA-cR		Aptamer LC-18 K_D = 38 nM Tumor Cells, Transformed Blood Vessels F-TGCCCGAACGCGAGTTGAGT TCCGAGAGCTCCGACTTCTT-cR		Aptamer LC-224 K_D = 96 nM Connective Tissues, Blood Vessels F-CCGGTAAATTCTCCTGACGCC GGGGTAAGTTTCTGAAATG-cR	
Aptamer-Associated Protein, Defined by MS Analyses	Binding Partner, Verified by Replacement with Correspondent Abs	Aptamer-Associated Protein, Defined by MS Analyses	Binding Partner, Verified by Replacement with Correspondent Abs	Aptamer-Associated Protein, Defined by MS Analyses	Binding Partner, Verified by Replacement with Correspondent Abs
Vimentin	–	histone H2B	± ± ±	hemoglobin subunit alpha/ beta	–
Prelamin-A/C; Lamin-A/C	–	actin cytoplasmic	–	actin cytoplasmic	+ + ±
Anexin A2, A5	–	glyceraldehyde-3-phosphate dehydrogenase	–	myosin-9	–
Actincytoplasmic	–	vimentin	+ ± –	keratintype I cytoskeletal	–
Neutrophil defensin1	± ± ±	prelamin-A/C; lamin-A/C	+ ± ±	neutrophil defensin1	–
Heat shock protein beta-1	–	pyruvate kinase isozymes M1/M2	–	glyceraldehyde-3-phosphate dehydrogenase	–
Pyruvate kinase isozymes M1/M2	–	hemoglobin subunits alpha/beta	–	tubulinalpha/ beta	–
Tubulin alpha/beta	± ± ±	neutrophil defensin 1	–	heat shock protein beta-1	–
		heat shock protein beta-1	–	pyruvate kinase isozymes M1/M2	–

The forward PCR primer (F), CTC CTC TGA CTG TAA CCA CG; the reverse-complement of the reverse PCR primer (cR), GCA TAG GTA GTC CAG AAG CC.

in Figure 1, IB4). Analyses of the fluorescence intensity (Figure 1, IB5) showed that aptamer's fluorescence in cells was more intense than that of anti-vimentin antibodies, potentially because other proteins beside vimentin associated with LC-18. Indeed, co-staining with anti-lamin antibodies revealed that LC-18 also bound to nuclear lamina. Hence, it could be concluded that aptamer LC-18 binds to nuclear lamina as well as vimentin in some tissues. Interestingly, lamin-A/C (P02545) and vimentin (P08670), according to the Protein Blast analyses, have 62% of query cover and 29% of identity. Hence, LC-18 potentially binds to the similar parts of these two proteins (Table S3). Vimentin, a major constituent of the intermediate filament family of proteins, is implicated in cancer initiation and progression, tumorigenesis, epithelial-to-mesenchymal transition, and invasion.[22–24] In lung cancers, vimentin expression was detected in moderately and well-differentiated adenocarcinoma and in giant cell carcinoma.[22,25] Lamins are the major component of the nuclear lamina, and changes in their expression can modulate cell proliferation, differentiation, as well as epithelial-mesenchymal transition and migration; all of these processes are important in cancer progression.[26,27] The presence of A-type lamins is a marker of differentiated tumors, with downregulation of A-type lamins in poorly differentiated tumors; hence, lamin A expression has been shown to be a marker of good or poor patient survival, depending on tumor subtype.[28,29] Furthermore, prelamin as a precursor of lamin A prevents cancer invasion.[23]

None of the investigated antibodies completely displaced LC-17 (Figure S4C). Only anti-tubulin alpha and neutrophil defensin 1 shifted the histograms to 51% and 46% accordingly (Figure 2, IIA and IIB). Tissue section experiments demonstrated LC-17 co-localization with anti-tubulin alpha antibody (Figure 2, IA), as shown by matching fluorescence peaks (Figure 2, IA1). In contrast, peaks for the fluorescence intensities of LC-17 and anti-neutrophil defensin antibodies did not overlap (Figure 2, IB5), concluding that the two ligands do not share the same epitope. Therefore, tubulin alpha has been verified as a protein associated with aptamer LC-17. Expression of different tubulin isotypes as well as their post-translational modifications has been correlated with poor prognosis and chemotherapy resistance and has been reported in various cancers.[30] Neutrophil defensin 28 increases in vitro migration and proliferation of epithelial cells through epidermal growth factor receptor activation and downstream signaling pathways.[31] Neutrophil defensins are overexpressed in several tumor types and associate with cancer invasiveness.[32]

The binding of aptamer LC-224 to blood vessels (Figure S5) was verified using anti-CD31 antibodies (Figure 3, IA and IIA). Indeed, this aptamer binds to blood vessel walls, but its binding epitopes are not the same as anti-CD31 antibodies, as observed from the fluorescence intensity profile (Figure 3, IA5). Moreover, LC-224 was not displaced with anti-CD31 antibody. Therefore, most probably, LC-224 does not bind to endothelial cells. Aptamer LC-224 was almost completely displaced only with anti-actin antibodies from all studied antibodies (Table 1; Figure S4B; Figure 3, IIB). Co-staining of tissue sections with anti-actin antibody and LC-224 also ensured co-localization of these two ligands. Actin is present in both healthy and transformed tissues, and its post-translational modifications in tumors can help LC-224 distinguish cancer cells from other cells. Indeed, analyzing mass spectra of actin purified using LC-224-coated magnetic particles from the samples of a lung adenocarcinoma patient revealed that all actin cytoplasmic 1 have a post-translational

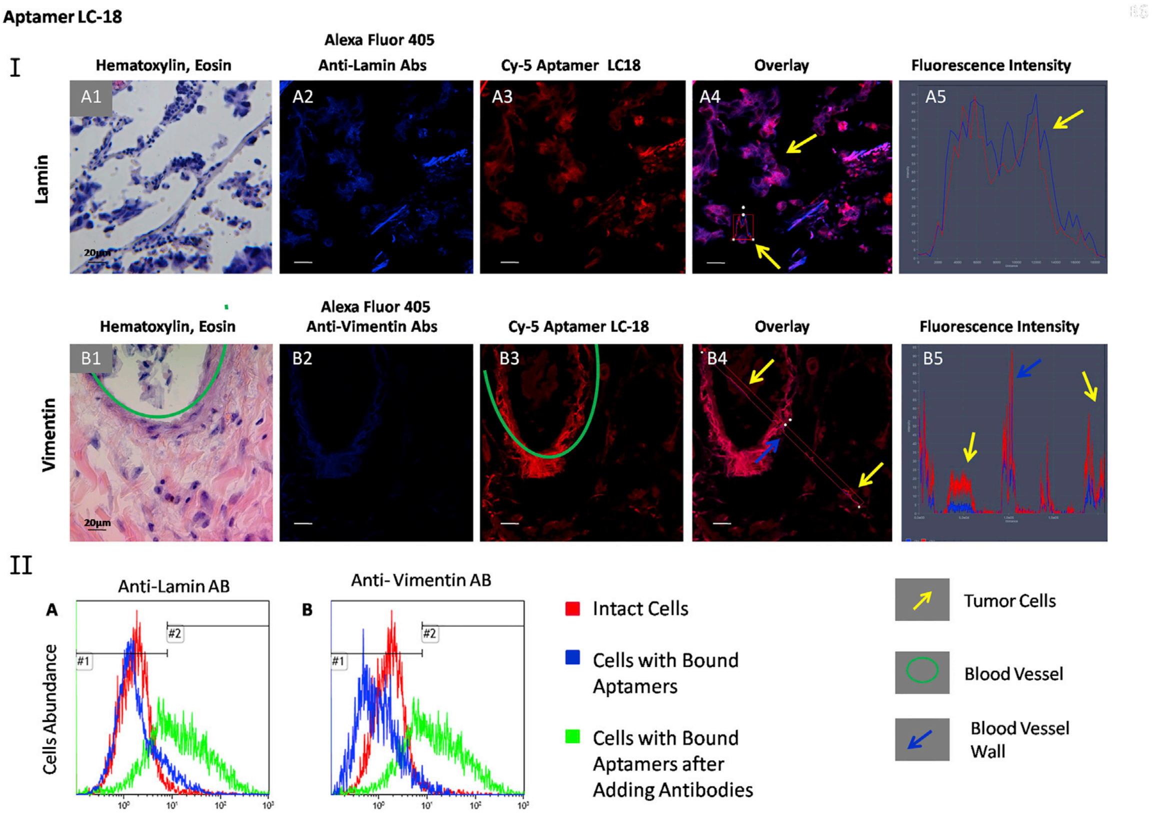

Figure 1. Verification of Lamin and Vimentin as LC-18-Associated Protein Targets

(I) Co-staining of the tissue sections with Alexa Fluor 405-labeled anti-lamin (A) and anti-vimentin (B) antibodies and Cy-5-labeled aptamer LC-18. (1) H&E staining of the adjacent section, (2) staining with correspondent Alexa Fluor 405-labeled antibody, (3) Cy-5-labeled aptamer LC-18, (4) overlay, and (5) histogram of fluorescence intensity are shown. Magnification, 40×. (II) Flow cytometry histograms indicating binding of aptamer LC-18 to lung adenocarcinoma cells and its replacement with anti-lamin (A) and anti-vimentin (B) antibodies. Red curve corresponds to intact lung adenocarcinoma cells, blue curve corresponds to cells bound with the aptamer LC-18 (50 nM), and green curve corresponds to the same sample after incubation with 2 ng μL^{-1} antibodies for 30 min with shaking at 37°C.

modification: histidine at position 73 is methylated (Figure S6). Actin is known as a promising prognostic biomarker and/or therapeutic target for metastatic lung adenocarcinoma.[33] The driving force for membrane protrusion is localized polymerization of submembrane actin filaments. Indeed, signal molecules linked to the actin cytoskeleton are upregulated in invasive and metastatic cancer cells.[34] Overexpression of actin in tumor cells of patients with lung adenocarcinomas resulted in enhanced distant metastasis and unfavorable prognosis. Actin downregulation impaired in vitro migration, invasion, clonogenicity, and transendothelial penetration of lung adenocarcinoma cells without affecting proliferation.[33]

To ensure aptamers could be used for therapeutic purposes, such as intraoperative cancer cell visualization, and to find their targets inside the tumor, we used aptahistochemical analyses of the fresh unprocessed tissues taken following surgery. We tested the aptamers for their ability to bind to various tumor structure elements in different parts of the tumor tissues, tumor margins (adjacent tissues), from four patients diagnosed with lung adenocarcinoma and healthy lung tissues taken as a control. Each piece of the same tumor part was divided into adjoined pieces, one of which was immediately stained with the DNA aptamers and the other with H&E dyes. This was done to ensure aptamer binding to certain tumor elements in the same tissue area. Aptamer staining was carried out according to the scheme depicted in Figure S1.

For the experiments, we used tissues from four adenocarcinoma patients and healthy lung. The first sample was poorly differentiated adenocarcinoma, nodular form with peribronchial growth with intraorganic metastases. The second sample was moderately differentiated adenocarcinoma with blood circulatory disorders, extensive necrosis, the germination of the wall of the bronchus, and infiltrative growth in the adjacent lung tissue. The third tissue sample was moderately differentiated invasive adenocarcinoma with increased vascularization. Patient 4, from whom we used a tumor and its margins, had metastatic poorly differentiated adenocarcinoma. Healthy lung tissue was kindly provided by the Krasnoyarsk Regional Clinical Pathological Anatomical Bureau. Tumor tissues were taken from patients who had undergone complete section of the lobe with the tumor. The lung cancer specimens were collected with the written informed

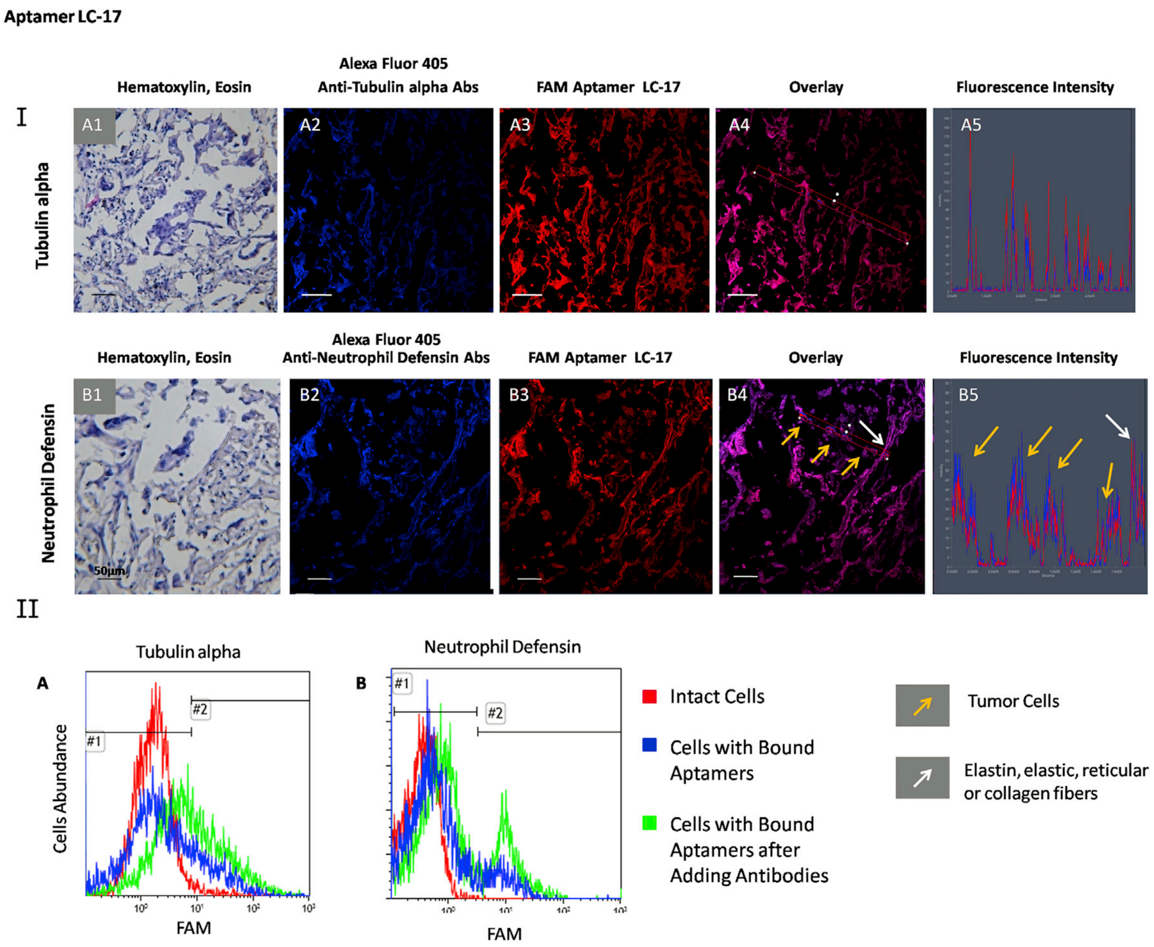

Figure 2. Verification of Tubulin Alpha and Neutrophil Defensin as LC-17-Associated Protein Targets

(I) Co-staining of the tissue sections with Alexa Fluor 405-labeled anti-tubulin alpha (A) and anti-neutrophil defensin (B) antibodies and Cy-5 labeled aptamer LC-17. (1) H&E staining of the adjacent section, (2) staining with correspondent Alexa Fluor 405-labeled antibody, (3) Cy-5-labeled aptamer LC-17, (4) overlay, and (5) histogram of fluorescence intensity are shown. Magnification, 40×. (II) Flow cytometry histograms indicating binding of aptamer LC-17 to lung adenocarcinoma cells and its replacement with anti-tubulin alpha (A) and anti-neutrophil defensin (B) antibodies. Red curve corresponds to intact lung adenocarcinoma cells, blue curve corresponds to cells bound with aptamer LC-17 (50 nM), and green curve corresponds to the same sample after incubation with 2 ng μL^{-1} antibodies for 30 min with shaking at 37°C.

consent of patients. Samples were transported to the laboratory within 1 hr of collection.

Aptahistochemical staining with aptamer LC-18 showed good binding, mostly to tumor cells. Tissue from the first patient was with a plurality of tumor cells (red arrows in Figure 4A, 1–3), with some of them concentrated around the gland (green circle in Figure 4A). The tissue of the second patient (Figure 4B) was necrotic, mostly with cell debris and few living tumor cells (red arrows in Figure 4B, 1–4) as well as destroyed elastic fibers (white arrows in Figure 4B, 2–4). Some spots resembled cells in the two-dimensional picture (Figure 4B, 2), but they were elongated when rotated in 3D (Figure 4B, 3), most likely indicative of damaged connective tissue. In the third adenocarcinoma sample, aptamer LC-18 bound to the tumor cells and elastic fibers of transformed tumor arteries (Figure 4C, 3). This was highly valuable because this aptamer did not bind to non-transformed blood vessels in healthy lung (Figure 4F, 2), tumor margins (Figure 4D, 2), and the tumor from the first patient (Figure 4A, 4). Currently, anti-angiogenic strategies like bevacizumab have developed into standard treatment options, and new drugs like tyrosine kinase inhibitors have shown optimistic results in phase II trials but failed to translate into positive results in phase III trials.[35] Thus, aptamer LC-18 is very important and can be used as a vascularization marker, which is significant not only for diagnostic purposes but also for targeting abnormal angiogenesis.

Aptamer LC-17 targeted reticular or collagen fibers (blue arrows in Figure 5A) and tumor cells (yellow arrows in Figure 5A). Importantly, this aptamer bound to some cells in tumor margins, but not to connective tissue (yellow arrows in Figure 5D, 2). These might be transformed cancer cells invading healthy adjacent lung tissue. In normal lung tissue from the healthy individual, the binding was not observed for aptamer LC-17 (Figure 5F, 2).

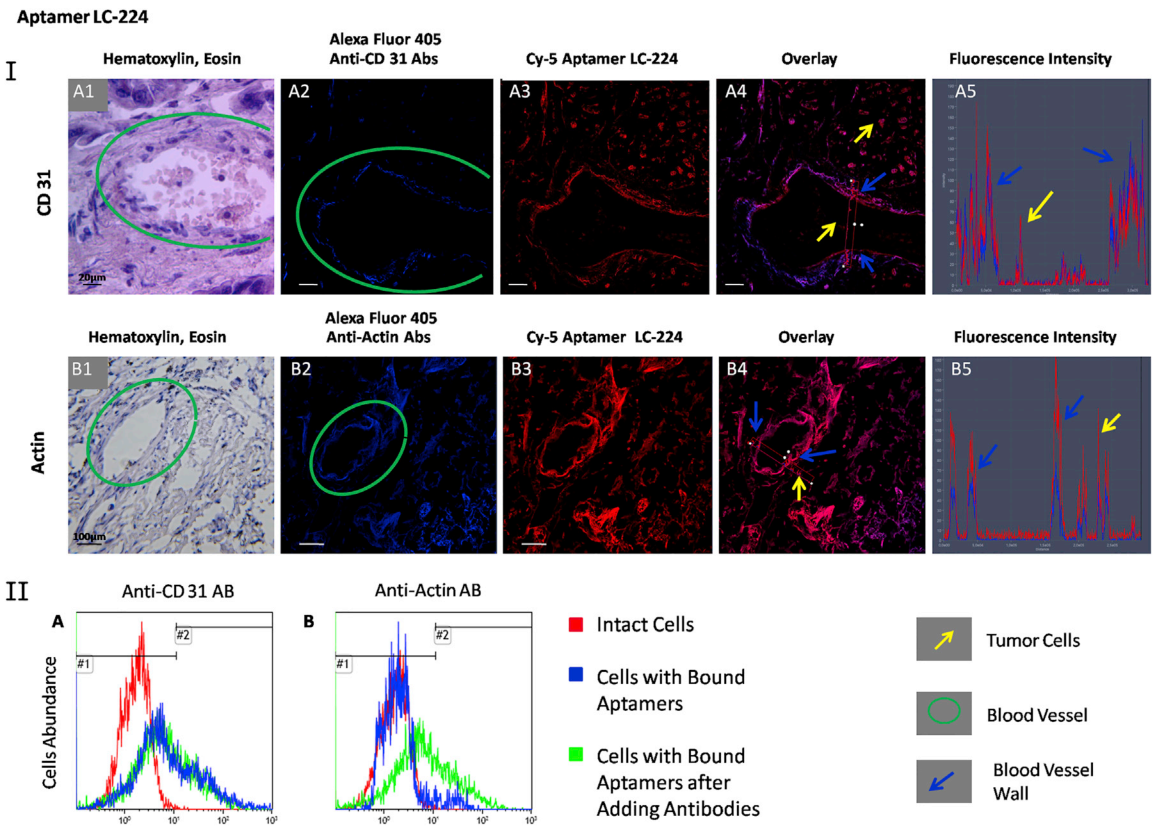

Figure 3. Verification of CD-31 and Actin as LC-224-Associated Protein Targets

(I) Co-staining of the tissue sections with Alexa Fluor 405-labeled anti-CD31 (A) and anti-actin (B) antibodies and Cy-5-labeled aptamer LC-224. (1) H&E staining of the adjacent section, (2) staining with correspondent Alexa Fluor 405-labeled antibody, (3) Cy-5-labeled aptamer LC-224, (4) overlay, and (5) histogram of fluorescence intensity are shown. Magnification, 40×. (II) Flow cytometry histograms indicating binding of the aptamer LC-224 to lung adenocarcinoma cells and its replacement with anti-CD31 (A) and anti-actin (B) antibodies. Red curve corresponds to intact lung adenocarcinoma cells, blue curve corresponds to cells bound with the aptamer LC-224 (50 nM), and green curve corresponds to the same sample after incubation with 2 ng μL^{-1} antibodies for 30 min with shaking at 37°C.

The next aptamer, LC-224, bound to connective tissues, arterial elastin, and elastic fibers, and it was found to slightly stain erythrocytes (Figure 6; Figure S6). Aptahistochemistry assays performed for different patients showed that LC-224 bound to elastic fibers (yellow arrows in Figure 6A, 1–3), most likely collagen fibers (yellow arrows in Figure 6B, 1–3), and elastic or reticular fibers (yellow arrows in Figure 6C, 1 and 2). Aptamer LC-224 did not bind to healthy lung tissues and tumor margin stroma, which indicates that the connective tissues of the tumor are transformed. Interestingly, this aptamer stained erythrocytes in tumor margins, but at the same time it did not bind to healthy erythrocytes in healthy lung tissue. This may be because the red blood cells may undergo transformation during cancerogenesis in lungs. If so, aptamer LC-224 may be a good and simple diagnostic tool.

Therefore, a combination of LC-17 and LC-18 could be useful to monitor the spread of cancer in lungs, because the cancer cells and transformed connective tissues can be visualized this way (Figure 7, IA and IB). Since we demonstrated that the pair of aptamers LC-17 and LC-18 are capable of binding to tumor stroma, we propose their use for tumor intraoperative visualization. This was modeled on the postoperative lung adenocarcinoma tissues using surgical fluorescent microscope OPMI Pentero (Carl Zeiss). Lung adenocarcinoma tissue was washed with PBS; afterwards, Brilliant Violet 650-labeled aptamers were applied to the surface and then washed for 2 min. One can see from Figure 7 (II) that the two aptamers stained cancer tissues and did not bind to necrotic tissues, blood vessels, and soot from smoke. Figure 7 (IIB and IID) demonstrates that LC-17 and LC-18 together could be used for tumor visualization in situ.

DISCUSSION

We show that the presented DNA aptamers have different binding to various lung tumor structures, such as elastic fibers, tumor cells, blood vessels, and elastin, all of which play important roles in tumor progression. In lung, elastic fibers are present in blood vessel walls, around alveolus, in the extracellular matrix, and in connective tissues;[36] therefore, it is likely that aptamers LC-17 and LC-224 have affinity to all of these structures. Moreover, these aptamers did not

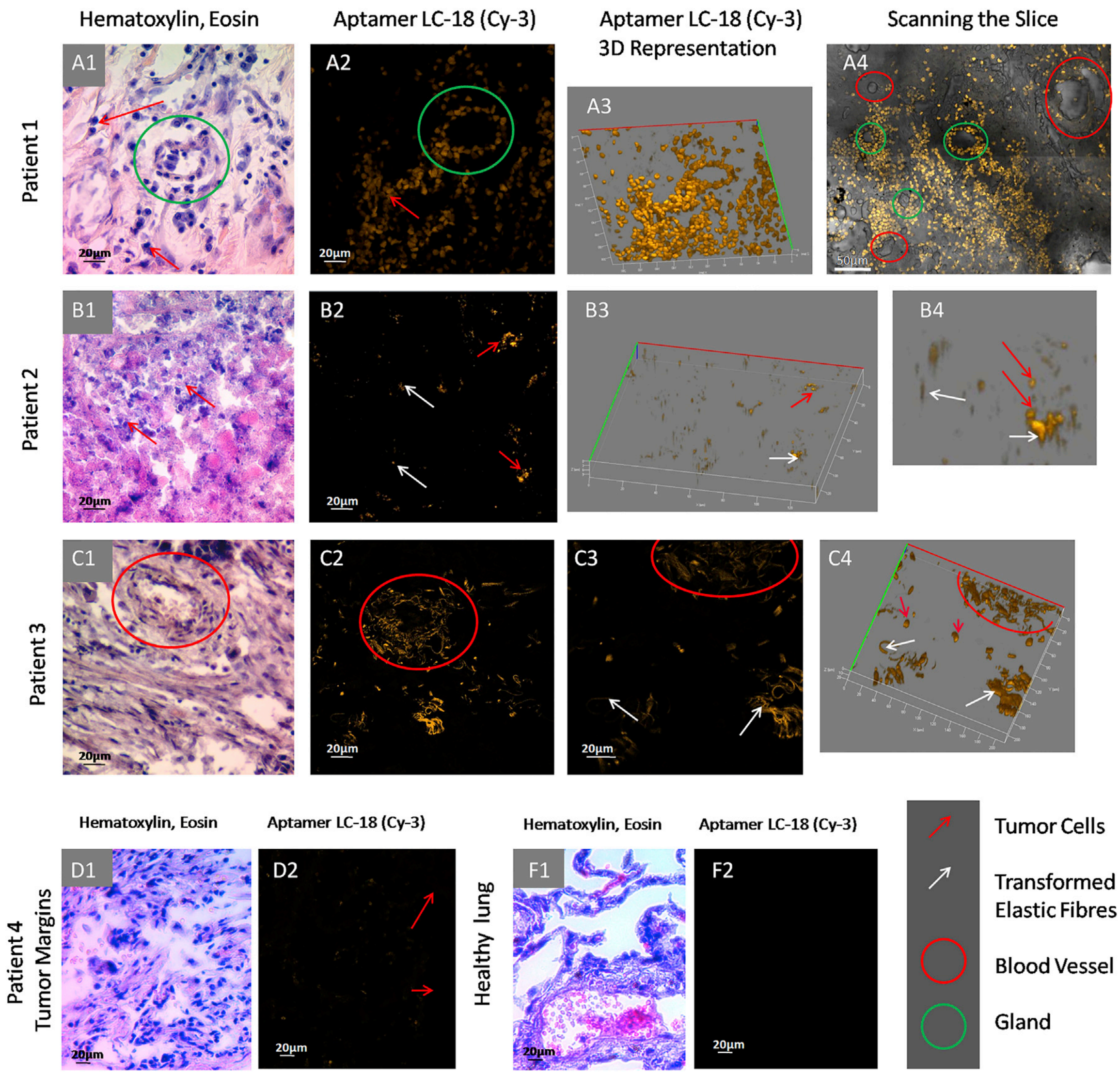

Figure 4. Binding of Aptamer LC-18 to Tumor Cells Transformed Elastic Fibers of the Blood Vessels in Tumor Tissues from Patients with Lung Adenocarcinoma

(A–F) Laser-scanning imaging of lung tissue sections stained with Cy-3-labeled LC-18 aptamers and light microscopy of lung tissue stained with H&E dyes. (A) Patient 1, (B) patient 2, (C) patient 3, (D1 and D2) tumor margins, and (F1 and F2) healthy lung tissues are shown. Magnification, 20× (A4, C1, C2, D1, and D2) and 40× (all others).

stain normal lung tissue, confirming that they only have affinity to elastic fibers that have been modified due to a malignant process (Figures 4, 5, and 6).

It is known that tumor vessels are strongly altered compared to normal, properly functioning vessels in healthy nonmalignant tissue. Tumor vessels are leaky, fenestrated, tortuous, dilated, and have chaotic irregular hierarchy branching patterns.[37] Recent studies have shown that endothelial cells lining the walls of blood vessels may appear modified as a result of reprogramming fibroblasts via SET-like protein and VEGF.[38] Fibroblasts are the most abundant cell type in connective tissues; they secrete extracellular matrix components and form the structural framework of tissues.[39] Peritumoral fibroblasts or cancer-associated fibroblasts (CAFs) are activated as a result of neoplastic processes, and they may be derived from pericytes, smooth muscle cells from the vasculature, and bone marrow-derived mesenchymal cells. One of the proposed sources of CAF origin is epithelial cells that achieve mesenchymal characteristics and become fibroblasts through an epithelial or endothelial mesenchymal transition process.[40–42] CAFs also are involved in inflammatory response and immune suppression in tumors, however, as expression profile depends on fibroblast location and functional status, the existence of general

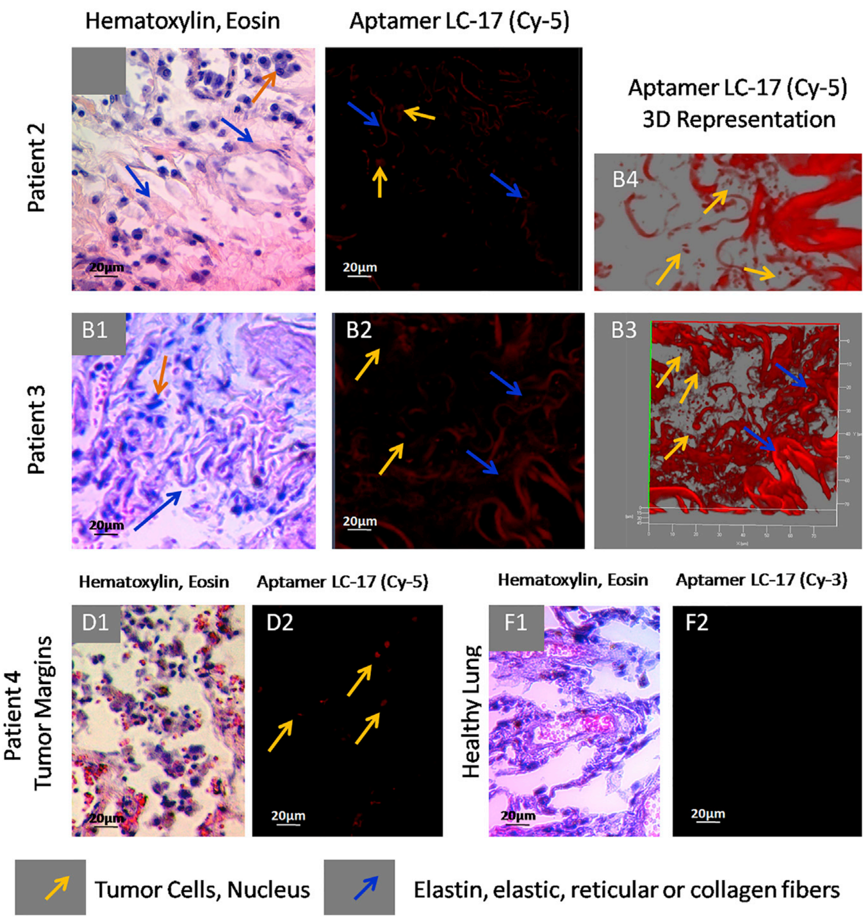

Figure 5. Binding of Aptamer LC-17 to Connective Tissues from Patients with Lung Adenocarcinoma, Including Elastic, Collagen Fibers, and Smooth Muscles

(A–D) Laser-scanning images of lung tissue sections stained with Cy-5-labeled LC-17 aptamer and light microscopy of lung tissue stained with H&E dyes. (A) Patient 1, (B) patient 2, (C) patient 3, (D1 and D2) tumor margins, and (F1 and F2) healthy lung tissues are shown. Magnification, 40×.

organ- and tissue-independent expression patterns is unclear.[43] These findings suggest that tumor-associated epithelial cells, modified connective tissues and vessels, and even certain immune cells present in tumor tissue may all have common markers that are functional for all of these structures, such as aptamer LC-17, which showed affinity to adenocarcinoma cells, glandular structures, probably transformed endothelial cells, and fibroblasts. Furthermore, each aptamer in our experiments binds to several tumor elements in tumor stroma and has several aptamer-associated proteins, with some of them in common for two or more aptamers. Thus, it may be concluded that the aptamers have an affinity to molecular markers involved in processes of transition between the structures of the tumor.

Aptamer LC-18 stained tumor cells as well as their nuclei and is associated with lamins, the main component of nuclear lamina. It is known that some cell surface proteins, such as tyrosine kinases receptors epidermal growth factor receptor (EGFR), ErbB-2, and fibroblast growth factor receptor (FGFR), are trafficked to the nucleus through different pathways.[44] Nuclear EGFR is associated with poor prognosis in several cancer types.[45–49] Cancer cells also utilize nuclear-cytoplasmic transport of tumor-suppressive proteins, such as retinoblastoma, APC, p53, BRAC1, FOXO proteins, INI1/hSNF5, galectin-3, Bok, nucleophosmin, RASSF2, Merlin, p21CIP, p27KIP1, N-WASP/FAK, estradiol receptor and Tob, drug targets topoisomerase I and IIa and BCR-ABL, and the molecular chaperone protein Hsp90, through the nuclear pore complex of a cell.[50] It is possible that certain cancer-related proteins translocated to the nuclei may be the targets of LC-17 and LC-18, since they have affinity to nuclei as well as to cell membranes, cytoplasm, and extracellular matrix.

Connective tissue-binding aptamers LC-224 and LC-17 were expected to bind to elastin or collagen; however, these proteins did not appear to be associated with them. It is possible that they also might have other additional non-protein targets, such as carbohydrates. Most importantly, by using the aptamers presented in this work, cancer and healthy tissue can be easily distinguished, and the histological type of the lung tumor can be further identified. Furthermore, using LC-18 and LC-224 it is possible to differentiate transformed blood vessels as well as abnormal vascularization. These aptamers can label cancer cells, abnormal blood vessels, and other transformed elements in tumor margins, which also could be very valuable. In situ visualization of the fresh unprocessed tumor tissues also can be accomplished using the pool of the two aptamers, LC-17 and LC-18. In prospect, these aptamers could be used as molecular carriers for targeted cancer therapy addressing abnormal blood vessels or tumor cells and intraoperative adenocarcinoma visualization.

Moreover, here we present a protocol for formalin-fixed, no paraffin-embedded cancer tissue with one aptamer, which is quick and easy to implement because it does not include procedures of tissue paraffinization and deparaffinization (Figure S1). With the thickness of the sections at 40 μm, it is easy to slice without the need of paraffin embedding, while at the same time the greater thickness does not decrease the quality of the confocal images. Slice thickness of 5 μm is optimal for staining with H&E dyes, which allows characterization of the structures of interest. The histopathology of freshly frozen tissues is carried out quicker and with less difficulty compared to the histopathology of formalin-fixed tissues.

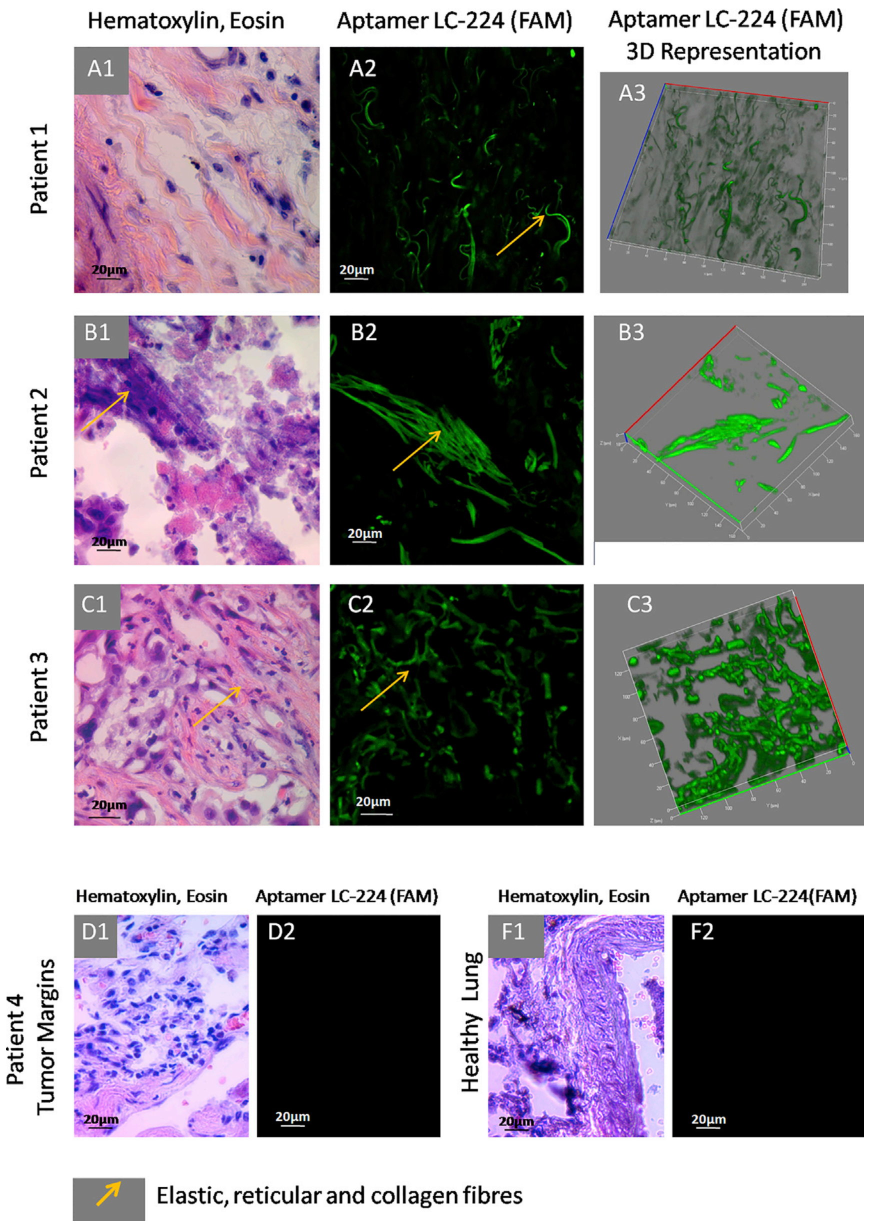

Figure 6. Binding of Aptamer LC-224 to Connective Tissues, Including Elastic, Reticular, and Collagen Fibers from Patients with Lung Adenocarcinoma

(A–D) Laser-scanning images of lung tissue sections stained with FAM-labeled LC-224 aptamers and light microscopy of lung tissue stained with H&E dyes. (A) Patient 1, (B) patient 2, (C) patient 3, (D1 and D2) tumor margins, and (F1 and F2) healthy lung tissues are shown. Magnification, 40×.

To conclude, we state that these aptamers could be utilized in immunohistochemistry-like assay (aptahistochemistry) for tumor tissue characterization, and they may act as the prognostic factors of the disease outcome as well as for tumor intraoperative visualization and targeted lung adenocarcinoma therapy.

MATERIALS AND METHODS

Tissue Section Staining with DNA Aptamers

This study and all experimental protocols were approved by the Local Committee on Ethics of the Krasnoyarsk Regional Clinical Cancer Center named after A.I. Kryzhanovsky and Krasnoyarsk State Medical University, and all methods were carried out in accordance with the approved guidelines. Blood for this study was taken from patients who had undergone complete lobectomy of the lung lobe with the tumor. The lung cancer specimens were collected with the written informed consent of patients. Samples were transported to the laboratory within 1 hr of collection.

Normal lung tissues were provided by Krasnoyarsk regional clinical pathology anatomical bureau and identified by the senior pathologist Sergey Bekuzarov.

Aptahistochemistry Assay

Sections from freshly frozen lung tissues were prepared from the tissues stained with the aptamers (Figure S2B). Fresh tissues obtained from surgery were cut with a scalpel into small pieces (~5 × 5 mm). The tissue pieces were washed three times with Dulbecco's phosphate-buffered saline (DPBS) with calcium and magnesium by shaking for 5 min, then non-specific binding was blocked with yeast RNA (1 ng μl^{-1}) (Sigma-Aldrich) by incubation for 2 hr at 6°C. Next, the tissue pieces were placed in 50 nM of the fluorescently labeled DNA aptamer solution in DPBS, incubated for 2 hr at 6°C, and washed with DPBS three times. Stained tissue pieces were frozen in liquid nitrogen and sliced into 5-μm sections by Microm HM525 Cryostat. One of each pair of adjacent sections was stained with H&E dye (Figure S2B). The tissue sections were analyzed by laser-scanning fluorescence microscopy using a Carl Zeiss LSM780 system.

Double Staining of the Tissue Sections with Antibodies and/or Aptamers

Tissue pieces were frozen in liquid nitrogen, sliced into 5-μm sections by Microm HM525 Cryostat, and placed on poly-lysine-coated glass slides. First, the non-specific binding of the antibodies was blocked by incubation of the sections with 10% BSA (Sigma-Aldrich) for 30 min, and then incubated with primary antibodies (0.001525 mg mL^{-1}) in a

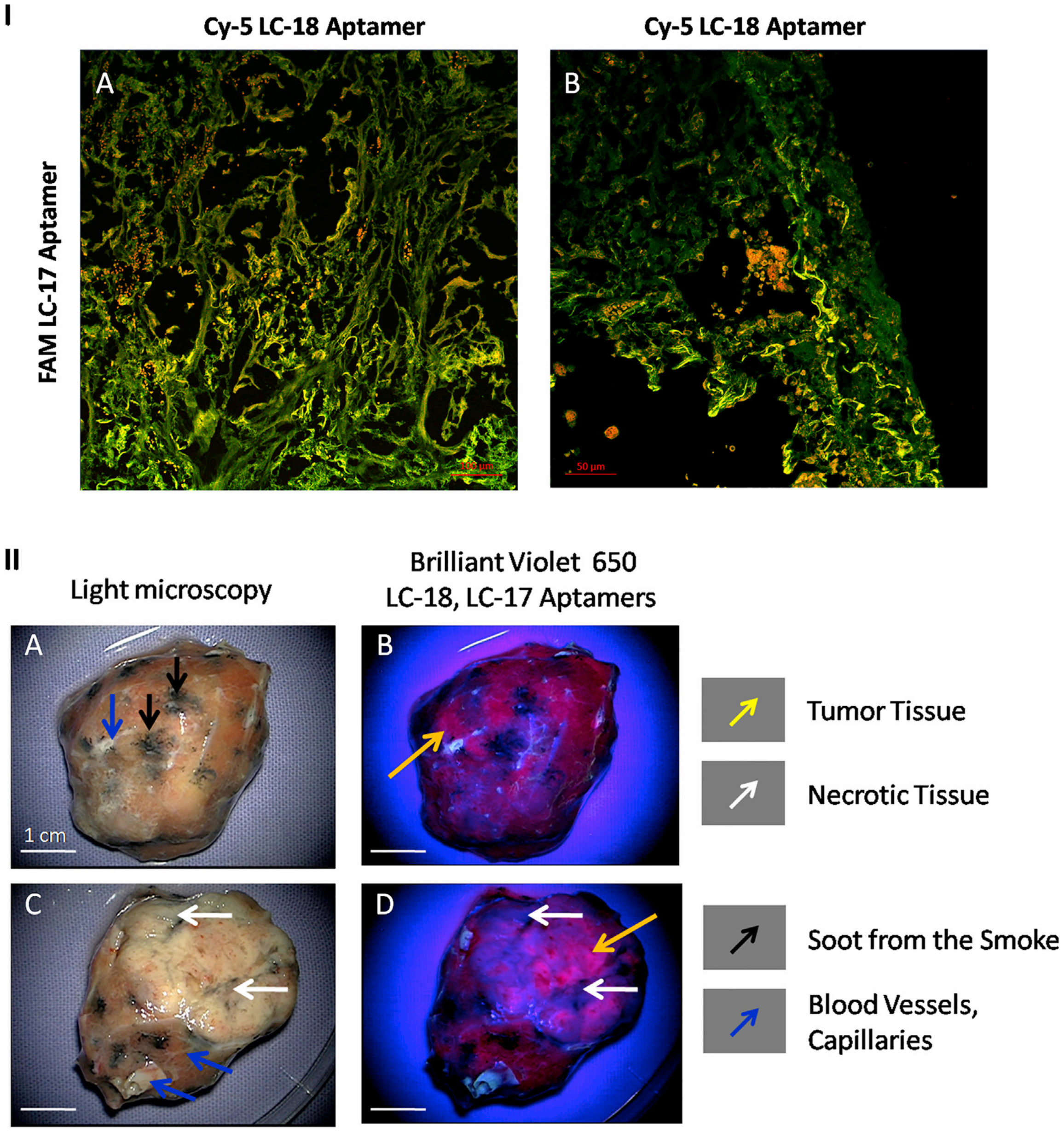

Figure 7. Postoperative Whole Tumor Imaging

(I) Co-staining of the lung adenocarcinoma tissue sections with FAM-labeled aptamer LC-17 and Cy-5 labeled aptamer LC-18 is shown. Magnification, 20× (A) and 40× (B). (II) In situ tumor visualization with brilliant violet 650-labeled LC-17 and LC-18 aptamers is shown.

humidified atmosphere for 1 hr, afterward with the secondary antibody labeled with Alexa Fluor 405 (0.0022 mg mL^{-1}) in a humidified atmosphere for 1 hr, and then washed three times with DPBS. Non-specific binding of the aptamers was blocked with yeast RNA (1 ng μl^{-1}) (Sigma-Aldrich) and then incubated with the 50 nM of the aptamers for 1 hr in a humidified atmosphere and washed with DPBS. Bio Mount mounting medium (Bio-Optica) was used to fix the sections.

Identification of the Aptamer-Associated Protein Biomarkers for Lung Adenocarcinoma

Identification of the proteins associated with aptamers LC-17, LC-18, and LC-224 was performed on the basis of the procedure described previously,[20,51] with some modifications described below. Here we used whole minced tumor tissues, in order to purify all tumor stroma proteins associated with the aptamers to increase the probability of the results, and, moreover, we increased the number

of patients in the study with adenocarcinoma to five to seven. Briefly, the proteins were identified after affinity purification from minced and lysed (0.1% [v/v] sodium deoxycholate in DPBS) tumor samples ($\sim$1 cm^3). Non-specific binding was blocked with masking yeast RNA (1 mg mL^{-1}) and 200 nM unlabeled single-stranded DNA (ssDNA)-library in DPBS. Afterward, incubation continued for another 30 min with 50 nM biotinylated aptamers. Proteins bound to the aptamers were purified using 1 mg Streptavidin MagneSphere Paramagnetic Particles (Promega). Samples for the mass spectrometry identification were prepared as described before. Shotgun mass spectrometric analysis of 10 μL protein-digest sample was performed by nanoflow ultra high-pressure liquid chromatography (DionexUltiMate 3000, Thermo Scientific) and tandem mass spectrometry with an OrbitrapFusion mass spectrometer (Thermo Scientific). Full-scan spectra were obtained using a Fourier transform mass analyzer with a resolution of 60,000. Fragment spectra of precursors were obtained for 4-s cycles by aion trapmass analyzer. Activation was performed by collision-induced dissociation. At the beginnings and ends of experimental sequences, BSA standard digest analyses were performed to estimate the quality of evolved results. Accuracy deviations for precursors were less than 4 ppm, which is appropriate for the external calibration standards. Database searches were done with Proteome Discoverer 1.3 software, Sequest search engine, and SwissProt database, and the label-free quantitative analyses were performed using MaxQuant 1.4 proteomic software. Triplicates of each experiment were performed. Experiments were repeated four times independently for all patients. Results from independent experiments were analyzed separately.

Flow Cytometric Replacement Analysis

Binding properties of the aptamers and their replacement with antibodies have been analyzed using flow cytometer FC-500 (Beckman Coulter). Lung adenocarcinoma tissues were washed with DPBS, minced, and pipetted gently in DPBS in order to remove cell clusters and obtain a homogeneous solution. Cell suspension was filtered through 70-μm filters; obtained cells were centrifuged at 3,000 × *g* for 5 min and washed three times with DPBS. Next, cells were pre-incubated with masking DNA (1 ng μL^{-1} salmon sperm DNA) for 30 min and then with 50 nM FAM-labeled aptamers from each pool of selection round or with synthetic aptamer sequences for 30 min at 25°C with shaking. Each sample contained 3 × 10^5 cells. LC cells pre-incubated with 1 ng μL^{-1} masking DNA and 50 nM FAM-labeled non-specific oligonucleotide CTC CTC TGA CTG TAA CCA CG $(AG)_{40}$ GCA TAG GTA GTC CAG AAG CC were used as a control. The measurements were carried out using flow cytometry. Afterwards, the samples were incubated with 2 ng μL^{-1} monoclonal antibodies (anti-actin, -lamin, -histonH2B, -neutrophil defensin, -tubulin alpha, -vimentin, -CD31, and –CEA; Abcam) for 30 min with shaking at 37°C, and flow cytometry was carried out again.

To evaluate the dissociation constants of the aptamers, cells were incubated with 1, 3, 5, 20, 70, 150, and 300 nM FAM-labeled aptamers. The measurements were carried out using flow cytometry. The data were analyzed with Kaluza 1.2 software. The dissociation constants were determined from the plots as half of the concentrations observed at maximum binding.

Whole-Tumor Visualization for Guided Surgery Purposes

Brilliant violet 650 Streptavidin (BioLegend) was attached to biotinylated aptamers in accordance with the manufacturer's protocol.

Whole resected unprocessed tumors, immediately after surgery, were washed with DPBS and sprayed with aptamers (1 μM) and immediately washed with DPBS. The images of the tumors before staining with aptamers were taken to ensure the absence of autofluorescence. Pictures were captured with a fluorescent microscope, OPMI Pentero (Carl Zeiss), with fluorescent module BLUE 400 (excitation at 400–410 nm and emission at 620–710 nm).

SUPPLEMENTAL INFORMATION

Supplemental Information includes eight figures and three tables and can be found with this article online at http://dx.doi.org/10.1016/j.omtn.2016.12.004.

AUTHOR CONTRIBUTIONS

G.S.Z., T.N.Z., M.V.B., and A.S.Z. conceived and designed the experiments. G.S.Z., T.I.I., V.L.G., Y.E.G., O.S.K., A.S.Z., I.V.G., A.A.B., and G.G.M. conducted the experiments. D.V.V. performed mathematical analyses. A.V.K., S.S.B., A.K.K., R.A.Z., M.M.P., and A.A.M. provided clinical samples and data. G.S.Z., A.S.Z., and A.G. wrote the paper. M.V.B. and A.S.Z. designed and supervised the work. All authors reviewed the manuscript.

CONFLICTS OF INTEREST

The authors declare no competing financial interests.

ACKNOWLEDGMENTS

The authors are grateful to all of the patients that participated in this research. This work was supported by the Ministry of Education and Science of the Russian Federation Federal Target Program 14.604.21.0105. Authors thank Dr. Marina A. Zenkova for her valuable ideas and Sergey Bayborodin and OPTEC Group for their help with laser-scanning fluorescence imaging. Microscopic analyses using Carl Zeiss LSM780 were carried out at the Multiple-Access Center for Microscopy of Biological Subjects (Institute of Cytology and Genetics) and LSM 800 in the Center for bioassay, nanotechnology and nanomaterials safety (Biotest-Nano) (Multiple-Access Center, Tomsk State University). The authors are grateful to Dr. Andrey Narodov for the help with tumor in situ visualization using surgical microscope OPMI Pentero in Krasnoyarsk Inter-District Ambulance Hospital named after N.S. Karpovich.

REFERENCES

1. Zeng, Z., Zhang, P., Zhao, N., Sheehan, A.M., Tung, C.H., Chang, C.C., and Zu, Y. (2010). Using oligonucleotide aptamer probes for immunostaining of formalin-fixed and paraffin-embedded tissues. Mod. Pathol. *23*, 1553–1558.

2. Zhao, Z., Xu, L., Shi, X., Tan, W., Fang, X., and Shangguan, D. (2009). Recognition of subtype non-small cell lung cancer by DNA aptamers selected from living cells. Analyst (Lond.) *134*, 1808–1814.

3. Pu, Y., Liu, Z., Lu, Y., Yuan, P., Liu, J., Yu, B., Wang, G., Yang, C.J., Liu, H., and Tan, W. (2015). Using DNA aptamer probe for immunostaining of cancer frozen tissues. Anal. Chem. *87*, 1919–1924.

4. Hanahan, D., and Weinberg, R.A. (2011). Hallmarks of cancer: the next generation. Cell *144*, 646–674.

5. Ischia, J., Patel, O., Bolton, D., Shulkes, A., and Baldwin, G.S. (2014). Expression and function of gastrin-releasing peptide (GRP) in normal and cancerous urological tissues. BJU Int. *113* (*Suppl 2*), 40–47.

6. Kim, M.J., Shin, H.C., Shin, K.C., and Ro, J.Y. (2013). Best immunohistochemical panel in distinguishing adenocarcinoma from squamous cell carcinoma of lung: tissue microarray assay in resected lung cancer specimens. Ann. Diagn. Pathol. *17*, 85–90.

7. Cairns, R.A., Harris, I.S., and Mak, T.W. (2011). Regulation of cancer cell metabolism. Nat. Rev. Cancer *11*, 85–95.

8. Wicki, A., Rochlitz, C., Orleth, A., Ritschard, R., Albrecht, I., Herrmann, R., Christofori, G., and Mamot, C. (2012). Targeting tumor-associated endothelial cells: anti-VEGFR2 immunoliposomes mediate tumor vessel disruption and inhibit tumor growth. Clin. Cancer Res. *18*, 454–464.

9. Rolny, C., Mazzone, M., Tugues, S., Laoui, D., Johansson, I., Coulon, C., Squadrito, M.L., Segura, I., Li, X., Knevels, E., et al. (2011). HRG inhibits tumor growth and metastasis by inducing macrophage polarization and vessel normalization through downregulation of PlGF. Cancer Cell *19*, 31–44.

10. Song, Y., Zhu, Z., An, Y., Zhang, W., Zhang, H., Liu, D., Yu, C., Duan, W., and Yang, C.J. (2013). Selection of DNA aptamers against epithelial cell adhesion molecule for cancer cell imaging and circulating tumor cell capture. Anal. Chem. *85*, 4141–4149.

11. Chen, H.W., Medley, C.D., Sefah, K., Shangguan, D., Tang, Z., Meng, L., Smith, J.E., and Tan, W. (2008). Molecular recognition of small-cell lung cancer cells using aptamers. ChemMedChem *3*, 991–1001.

12. Kunii, T., Ogura, S., Mie, M., and Kobatake, E. (2011). Selection of DNA aptamers recognizing small cell lung cancer using living cell-SELEX. Analyst (Lond.) *136*, 1310–1312.

13. Li, L., Zhao, H., Chen, Z., Mu, X., and Guo, L. (2011). Aptamer biosensor for label-free square-wave voltammetry detection of angiogenin. Biosens. Bioelectron. *30*, 261–266.

14. Souada, M., Piro, B., Reisberg, S., Anquetin, G., Noël, V., and Pham, M.C. (2015). Label-free electrochemical detection of prostate-specific antigen based on nucleic acid aptamer. Biosens. Bioelectron. *68*, 49–54.

15. Zhang, K., Sefah, K., Tang, L., Zhao, Z., Zhu, G., Ye, M., Sun, W., Goodison, S., and Tan, W. (2012). A novel aptamer developed for breast cancer cell internalization. ChemMedChem *7*, 79–84.

16. Sefah, K., Bae, K.M., Phillips, J.A., Siemann, D.W., Su, Z., McClellan, S., Vieweg, J., and Tan, W. (2013). Cell-based selection provides novel molecular probes for cancer stem cells. Int. J. Cancer *132*, 2578–2588.

17. Shi, H., He, X., Wang, K., Wu, X., Ye, X., Guo, Q., Tan, W., Qing, Z., Yang, X., and Zhou, B. (2011). Activatable aptamer probe for contrast-enhanced in vivo cancer imaging based on cell membrane protein-triggered conformation alteration. Proc. Natl. Acad. Sci. USA *108*, 3900–3905.

18. Herrmann, A., Priceman, S.J., Swiderski, P., Kujawski, M., Xin, H., Cherryholmes, G.A., Zhang, W., Zhang, C., Lahtz, C., Kowolik, C., et al. (2014). CTLA4 aptamer delivers STAT3 siRNA to tumor-associated and malignant T cells. J. Clin. Invest. *124*, 2977–2987.

19. Hicke, B.J., Stephens, A.W., Gould, T., Chang, Y.F., Lynott, C.K., Heil, J., Borkowski, S., Hilger, C.S., Cook, G., Warren, S., and Schmidt, P.G. (2006). Tumor targeting by an aptamer. J. Nucl. Med. *47*, 668–678.

20. Zamay, G.S., Kolovskaya, O.S., Zamay, T.N., Glazyrin, Y.E., Krat, A.V., Zubkova, O., Spivak, E., Wehbe, M., Gargaun, A., Muharemagic, D., et al. (2015). Aptamers selected to postoperative lung adenocarcinoma detect circulating tumor cells in human blood. Mol. Ther. *23*, 1486–1496.

21. Yamaguchi, H., and Condeelis, J. (2007). Regulation of the actin cytoskeleton in cancer cell migration and invasion. Biochim. Biophys. Acta *1773*, 642–652.

22. Havel, L.S., Kline, E.R., Salgueiro, A.M., and Marcus, A.I. (2015). Vimentin regulates lung cancer cell adhesion through a VAV2-Rac1 pathway to control focal adhesion kinase activity. Oncogene *34*, 1979–1990.

23. Kidd, M.E., Shumaker, D.K., and Ridge, K.M. (2014). The role of vimentin intermediate filaments in the progression of lung cancer. Am. J. Respir. Cell Mol. Biol. *50*, 1–6.

24. Richardson, F., Young, G.D., Sennello, R., Wolf, J., Argast, G.M., Mercado, P., Davies, A., Epstein, D.M., and Wacker, B. (2012). The evaluation of E-Cadherin and vimentin as biomarkers of clinical outcomes among patients with non-small cell lung cancer treated with erlotinib as second- or third-line therapy. Anticancer Res. *32*, 537–552.

25. Upton, M.P., Hirohashi, S., Tome, Y., Miyazawa, N., Suemasu, K., and Shimosato, Y. (1986). Expression of vimentin in surgically resected adenocarcinomas and large cell carcinomas of lung. Am. J. Surg. Pathol. *10*, 560–567.

26. Chow, K.H., Factor, R.E., and Ullman, K.S. (2012). The nuclear envelope environment and its cancer connections. Nat. Rev. Cancer *12*, 196–209.

27. Friedl, P., Wolf, K., and Lammerding, J. (2011). Nuclear mechanics during cell migration. Curr. Opin. Cell Biol. *23*, 55–64.

28. Machiels, B.M., Ramaekers, F.C., Kuijpers, H.J., Groenewoud, J.S., Oosterhuis, J.W., and Looijenga, L.H. (1997). Nuclear lamin expression in normal testis and testicular germ cell tumours of adolescents and adults. J. Pathol. *182*, 197–204.

29. Foster, C.R., Przyborski, S.A., Wilson, R.G., and Hutchison, C.J. (2010). Lamins as cancer biomarkers. Biochem. Soc. Trans. *38*, 297–300.

30. Parker, A.L., Kavallaris, M., and McCarroll, J.A. (2014). Microtubules and their role in cellular stress in cancer. Front. Oncol. *4*, 153.

31. Aarbiou, J., Verhoosel, R.M., Van Wetering, S., De Boer, W.I., Van Krieken, J.H., Litvinov, S.V., Rabe, K.F., and Hiemstra, P.S. (2004). Neutrophil defensins enhance lung epithelial wound closure and mucin gene expression in vitro. Am. J. Respir. Cell Mol. Biol. *30*, 193–201.

32. Holterman, D.A., Diaz, J.I., Blackmore, P.F., Davis, J.W., Schellhammer, P.F., Corica, A., Semmes, O.J., and Vlahou, A. (2006). Overexpression of alpha-defensin is associated with bladder cancer invasiveness. Urol. Oncol. *24*, 97–108.

33. Lee, H.W., Park, Y.M., Lee, S.J., Cho, H.J., Kim, D.H., Lee, J.I., Kang, M.S., Seol, H.J., Shim, Y.M., Nam, D.H., et al. (2013). Alpha-smooth muscle actin (ACTA2) is required for metastatic potential of human lung adenocarcinoma. Clin. Cancer Res. *19*, 5879–5889.

34. Sahai, E. (2005). Mechanisms of cancer cell invasion. Curr. Opin. Genet. Dev. *15*, 87–96.

35. Hilbe, W., Manegold, C., and Pircher, A. (2012). Targeting angiogenesis in lung cancer - pitfalls in drug development. Transl. Lung Cancer Res. *1*, 122–128.

36. Kielty, C.M., Sherratt, M.J., and Shuttleworth, C.A. (2002). Elastic fibres. J. Cell Sci. *115*, 2817–2828.

37. Seo, B.R., Delnero, P., and Fischbach, C. (2014). In vitro models of tumor vessels and matrix: engineering approaches to investigate transport limitations and drug delivery in cancer. Adv. Drug Deliv. Rev. *69–70*, 205–216.

38. Margariti, A., Winkler, B., Karamariti, E., Tsai, T., Zeng, L., Hu, Y., and Xu, Q. (2011). Direct reprogramming fibroblasts into endothelial cells. Heart *97*, e7.

39. Gabbiani, G., Ryan, G.B., and Majne, G. (1971). Presence of modified fibroblasts in granulation tissue and their possible role in wound contraction. Experientia *27*, 549–550.

40. Bremnes, R.M., Dønnem, T., Al-Saad, S., Al-Shibli, K., Andersen, S., Sirera, R., Camps, C., Marinez, I., and Busund, L.T. (2011). The role of tumor stroma in cancer progression and prognosis: emphasis on carcinoma-associated fibroblasts and non-small cell lung cancer. J. Thorac. Oncol. *6*, 209–217.

41. Cirri, P., and Chiarugi, P. (2011). Cancer associated fibroblasts: the dark side of the coin. Am. J. Cancer Res. *1*, 482–497.

42. Radisky, D.C., Kenny, P.A., and Bissell, M.J. (2007). Fibrosis and cancer: do myofibroblasts come also from epithelial cells via EMT? J. Cell. Biochem. *101*, 830–839.

43. Silzle, T., Randolph, G.J., Kreutz, M., and Kunz-Schughart, L.A. (2004). The fibroblast: sentinel cell and local immune modulator in tumor tissue. Int. J. Cancer *108*, 173–180.

44. Wang, Y.N., Lee, H.H., Lee, H.J., Du, Y., Yamaguchi, H., and Hung, M.C. (2012). Membrane-bound trafficking regulates nuclear transport of integral epidermal growth factor receptor (EGFR) and ErbB-2. J. Biol. Chem. *287*, 16869–16879.

45. Hadzisejdić, I., Mustać, E., Jonjić, N., Petković, M., and Grahovac, B. (2010). Nuclear EGFR in ductal invasive breast cancer: correlation with cyclin-D1 and prognosis. Mod. Pathol. *23*, 392–403.

46. Hoshino, M., Fukui, H., Ono, Y., Sekikawa, A., Ichikawa, K., Tomita, S., Imai, Y., Imura, J., Hiraishi, H., and Fujimori, T. (2007). Nuclear expression of phosphorylated EGFR is associated with poor prognosis of patients with esophageal squamous cell carcinoma. Pathobiology *74*, 15–21.

47. Lo, H.W., Xia, W., Wei, Y., Ali-Seyed, M., Huang, S.F., and Hung, M.C. (2005). Novel prognostic value of nuclear epidermal growth factor receptor in breast cancer. Cancer Res. *65*, 338–348.

48. Psyrri, A., Egleston, B., Weinberger, P., Yu, Z., Kowalski, D., Sasaki, C., Haffty, B., Rimm, D., and Burtness, B. (2008). Correlates and determinants of nuclear epidermal growth factor receptor content in an oropharyngeal cancer tissue microarray. Cancer Epidemiol. Biomarkers Prev. *17*, 1486–1492.

49. Xia, W., Wei, Y., Du, Y., Liu, J., Chang, B., Yu, Y.L., Huo, L.F., Miller, S., and Hung, M.C. (2009). Nuclear expression of epidermal growth factor receptor is a novel prognostic value in patients with ovarian cancer. Mol. Carcinog. *48*, 610–617.

50. Turner, J.G., Dawson, J., and Sullivan, D.M. (2012). Nuclear export of proteins and drug resistance in cancer. Biochem. Pharmacol. *83*, 1021–1032.

51. Zamay, T.N., Kolovskaya, O.S., Glazyrin, Y.E., Zamay, G.S., Kuznetsova, S.A., Spivak, E.A., Wehbe, M., Savitskaya, A.G., Zubkova, O.A., Kadkina, A., et al. (2014). DNA-aptamer targeting vimentin for tumor therapy in vivo. Nucleic Acid Ther. *24*, 160–170.

OMTN, Volume *6*

Supplemental Information

DNA Aptamers for the Characterization of Histological Structure of Lung Adenocarcinoma

Galina S. Zamay, Tatiana I. Ivanchenko, Tatiana N. Zamay, Valentina L. Grigorieva, Yury E. Glazyrin, Olga S. Kolovskaya, Irina V. Garanzha, Andrey A. Barinov, Alexey V. Krat, Gleb G. Mironov, Ana Gargaun, Dmitry V. Veprintsev, Sergey S. Bekuzarov, Andrey K. Kirichenko, Ruslan A. Zukov, Marina M. Petrova, Andrey A. Modestov, Maxim V. Berezovski, and Anna S. Zamay

Table S1. Sequences of aptamers. Where F: CTC CTC TGA CTG TAA CCA CG (the forward PCR primer) and cR:GCA TAG GTA GTC CAG AAG CC (the reverse-complement of the reverse PCR primer).

Aptamer	Sequence
LC-17	F-CTTTTGTCTTTAGCCGAATTTTACTAAGCCGGGCTGATCA-cR
LC-18	F-TGCCCGAACGCGAGTTGAGTTCCGAGAGCTCCGACTTCTT-cR
LC-224	F-CCGGTAAATTCTCCTGACGCCGGGGTAAGTTTCTGAAATG-cR
LC-2107	F-CGCGGTGAAGGGTATATCCACTGCGTCCCGTGCCGTCGGT-cR
LC-2108	F-CCCAGAGTCAGTGCGGCCCTTCCTTACAGTTTACCCCCGA-cR
LC-29	F-ATACCAAAGTCGTCTCCGCTCCGGTTGCACAACGAAGTTC-cR

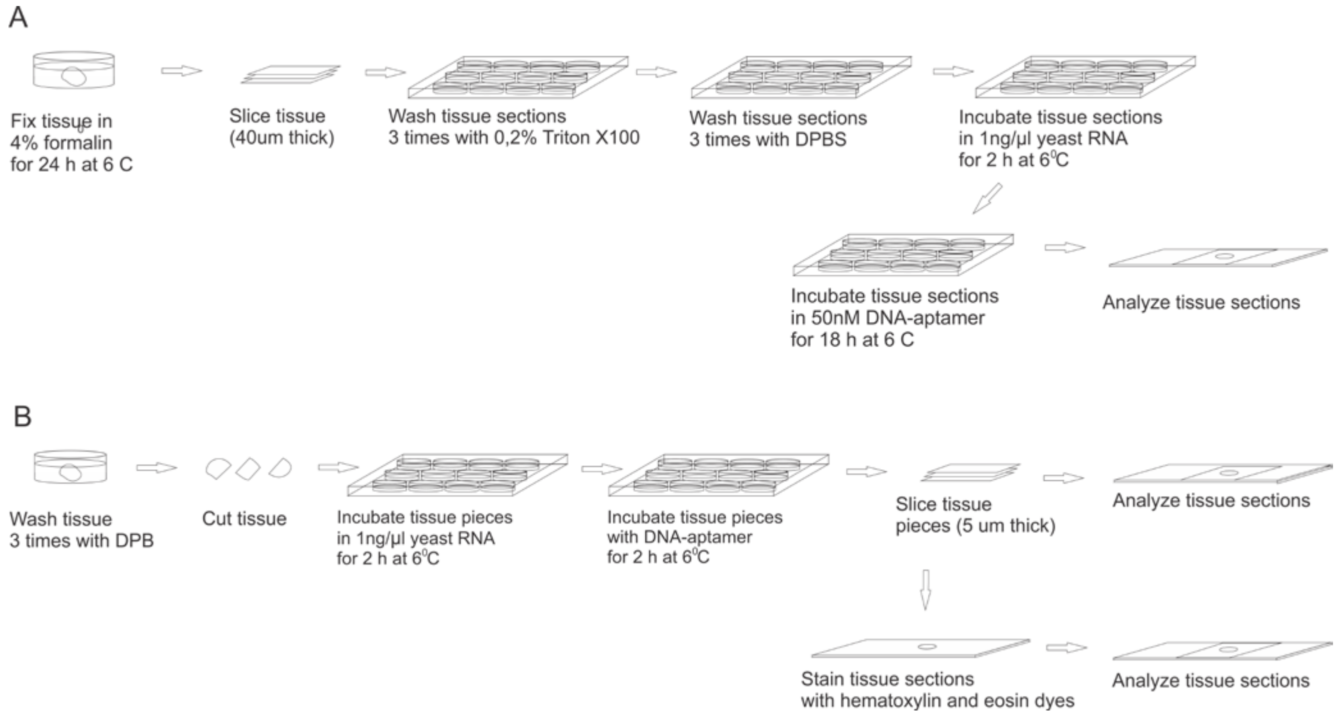

Figure S1. An overview of lung cancer tissue aptamer staining. (A) Tissue was fixed in 4% formalin, sliced, and washed 3 times with 0.2% Triton X-100 in DPBS. This was followed by incubation with 1 ng μL^{-1} yeast RNA in DPBS, incubation with 50 nM of a fluorescently labeled DNA-aptamer and analysis by microscopy. (B) Tissue was washed 3 times with DPBS and cut into smaller pieces. Next, the tissue pieces were incubated in 1 ngμL^{-1} yeast RNA in DPBS, and then incubated in sequence with DNA-aptamers. After washing, tissue pieces were sliced one of two adjacent slices were stained with hematoxylin and eosin dyes. All tissue sections were finally analyzed using fluorescent (or for better results laser scanning)and light microscopy.

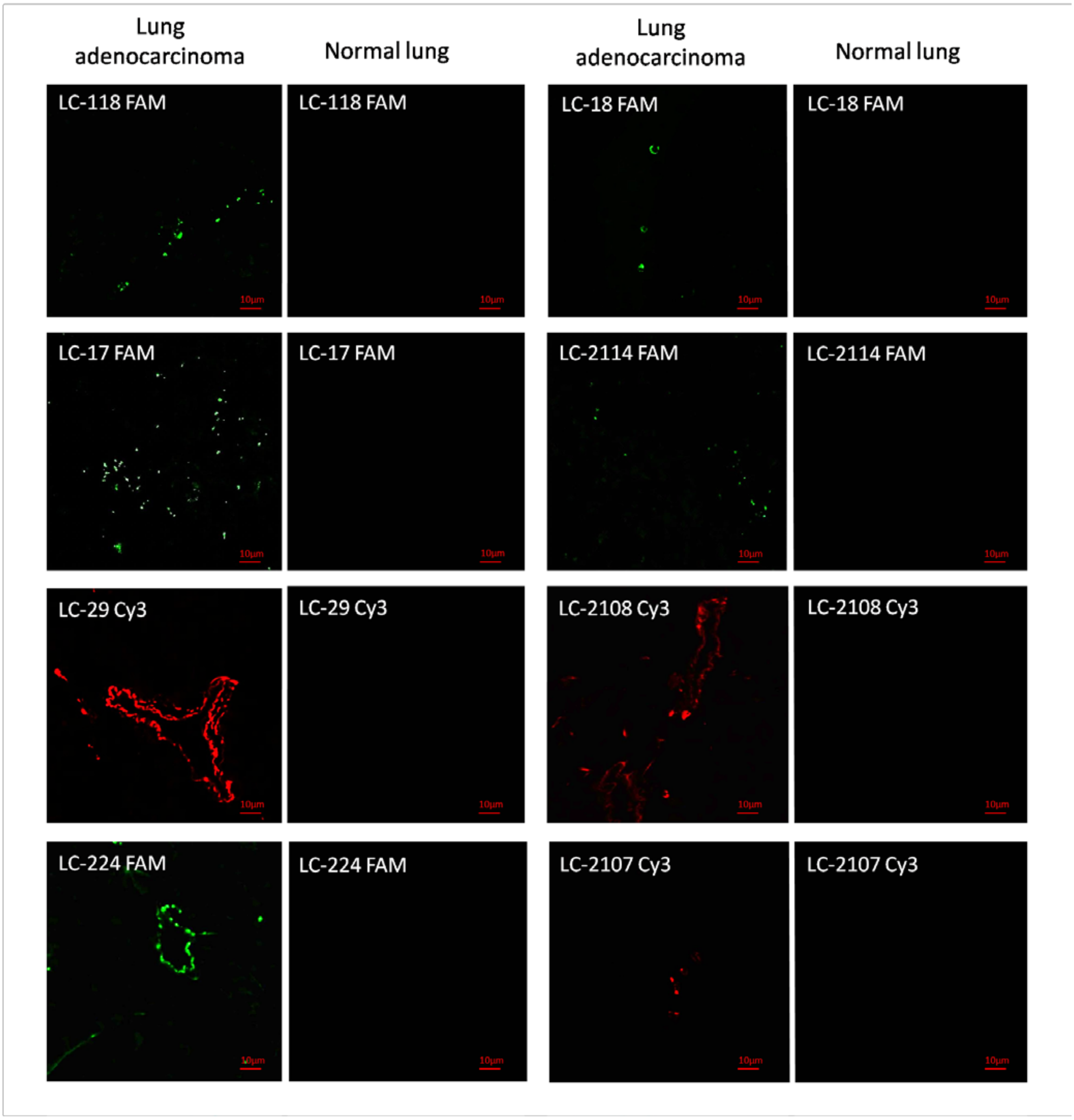

Figure S2. Cells (A) and blood vessels (C) alone and together (B) in lung adenocarcinoma tissue visualized with the following aptamers: LC-17 (A1, A2, B), LC-29 (C1, C3), LC-2108 (C2, C3). Laser scanning imaging (40X).

Table S2. Tissue structures in lung adenocarcinomatargeted by the aptamers.

	Aptamers to Lung Adenocarcinoma							
Targeted Structure	LC-17	LC-18	LC-29	LC-118	LC-224	LC-2107	LC-2108	LC-2114
Tumor Cells	+++	+++	+++	+++	--+	+++	---	+++
Connective Tissues	+++	---	---	---	+++	---	---	---
Blood Vessels	---	--+	---	---	+++	---	+++	---
Fibers	+++	---	---	---	+++	---	+++	---
Glandular Structures	---	+++	+++	---	---	---	---	---
Bronho-Alveolar Structures	---	---	+++	---	---	+++	---	---
Necrotic Tissues	---	+++	---	+++	---	---	---	---
Erythrocytes	---	---	---	---	+++	---	+++	---
Healthy LungCells	---	---	---	---	---	---	---	---
Healthy Connective Tissues	---	---	---	---	---	---	---	---
Cells in Tumor Margins	-+	++	+-	++	+-	++	-+	++

+ or - indicate binding of the aptamer to correspondent structure element in lung adenocarcinoma tissues derived from one patient.

Table S3. Pairwise alignment of lamin-A/C (P02545) and vimentin (P08670) protein sequence performed using program Protein Blast.

Range 1: 99 to 416 GenPept Graphics ▼ Next Match ▲ Previous Match

Score	Expect	Method	Identities	Positives	Gaps
131 bits(330)	4e-37	Compositional matrix adjust.	107/367(29%)	194/367(52%)	49/367(13%)

```
Query  27   TRLQEKEDLQELNDRLAVYIDRVRSLETENAGLRLRITESEEVVSREVSGIKAAYEAELG  86
            TR  EK +LQELNDR A YID+VR LE +N   ++ + E E++  +  S +   YE E+
Sbjct  99   TRTNEKVELQELNDRFANYIDKVRFLEQQN---KILLAELEQLKGQGKSRLGDLYEEEMR  155

Query  87   DARKTLDSVAKERARLQLELSKVREEFKELKARNTKKEGDLIAAQARLKDLEALLNSKEA  146
            + R+ +D +  ++AR+++E   + E+   L                R K  E +L  +EA
Sbjct  156  ELRRQVDQLTNDKARVEVERDNLAEDIMRL----------------REKLQEEMLQREEA  199

Query  147  ALSTALSEKRTLEGELHDLRGQVAKLEAALGEAKKQLQDEMLRRVDAENRLQTMKEELDF  206
                                      E  L   ++ + +  L R+D E ++++++EE+ F
Sbjct  200  --------------------------ENTLQSFRQDVDNASLARLDLERKVESLQEEIAF  233

Query  207  QKNIYSEELRETKRRHETRLVEIDNGKQREFESRLADALQELRAQHEDQVEQYKKELEKT  266
             K ++ EE++E + + + + V+ID    +     L  AL+++R Q+E    +  +E E+
Sbjct  234  LKKLHEEEIQELQAQIQEQHVQIDVDVSK---PDLTAALRDVRQQYESVAAKNLQEAEEW  290

Query  267  YSAKLDNARQSAERNSNLVGAAHEELQQSRIRIDSLSAQLSQLQKQLAAKEAKLRDLEDS  326
            Y +K  +  ++A RN++ +  A +E  + R ++ SL+ ++  L+    + E ++R++E++
Sbjct  291  YKSKFADLSEAANRNNDALRQAKQESTEYRRQVQSLTCEVDALKGTNESLERQMREMEEN  350

Query  327  LARERDTSRRLLAEKEREMAEMRARMQQQLDEYQELLDIKLALDMEIHAYRKLLEGEEER  386
             A E    +  +   + E+  M+  M + L EYQ+LL++K+ALD+EI  YRKLLEGEE R
Sbjct  351  FAVEAANYQDTIGRLQDEIQNMKEEMARHLREYQDLLNVKMALDIEIATYRKLLEGEESR  410

Query  387  LRLSPSP  393
            + L P P
Sbjct  411  ISL-PLP  416
```

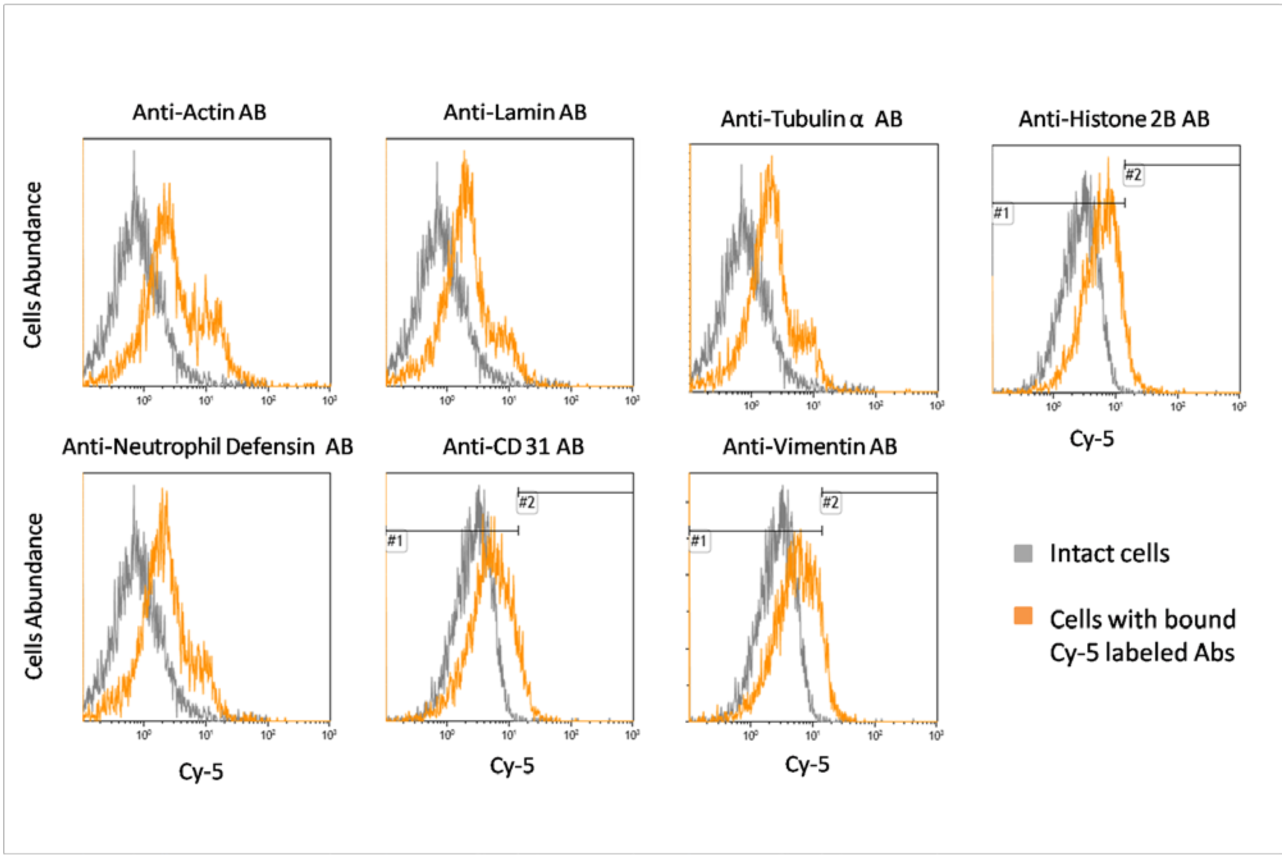

Figure S3. Flow cytometry histograms indicating binding of different antibodies (which have been used for aptamer replacement analyses) to lung adenocarcinoma cells. Gray curve corresponds to intact lung adenocarcinoma cells, orange - cells bound with Cy-5 labeled antibodies.

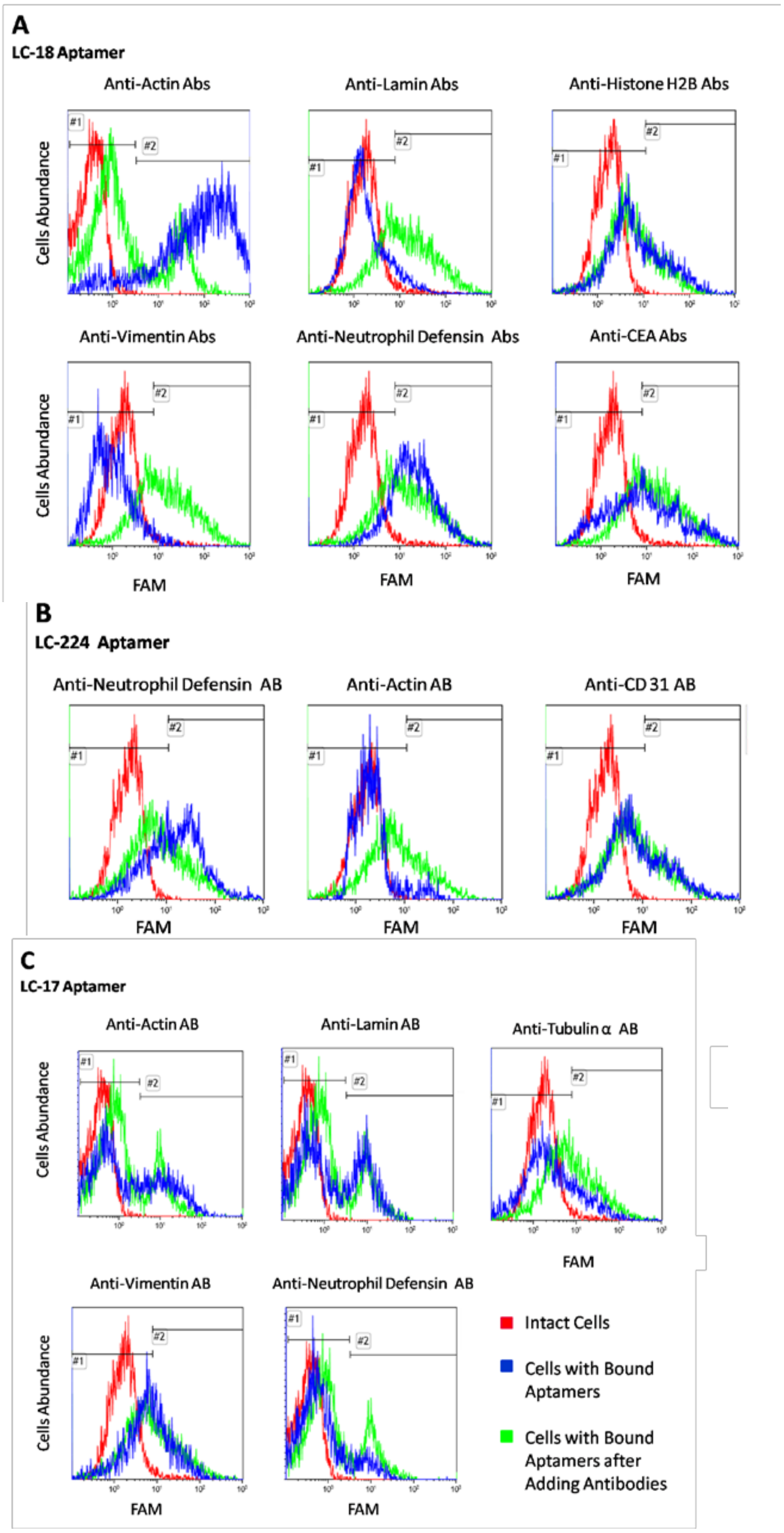

Figure S4. Flow cytometry histograms indicating binding of the aptamers LC-18 (A), LC-17 (B), LC-224 (C) to lung adenocarcinoma cells and its replacement with different antibodies (to correspondent proteins which were identified as aptamer-associated targets). Blue curve corresponds to intact lung adenocarcinoma cells, green to cells bound with the aptamer LC-18 (50nM) and blue - the same sample after incubation with 2 ng μL^{-1}Cy-5 labeled antibodies for 30 min with shakingat 37°C.

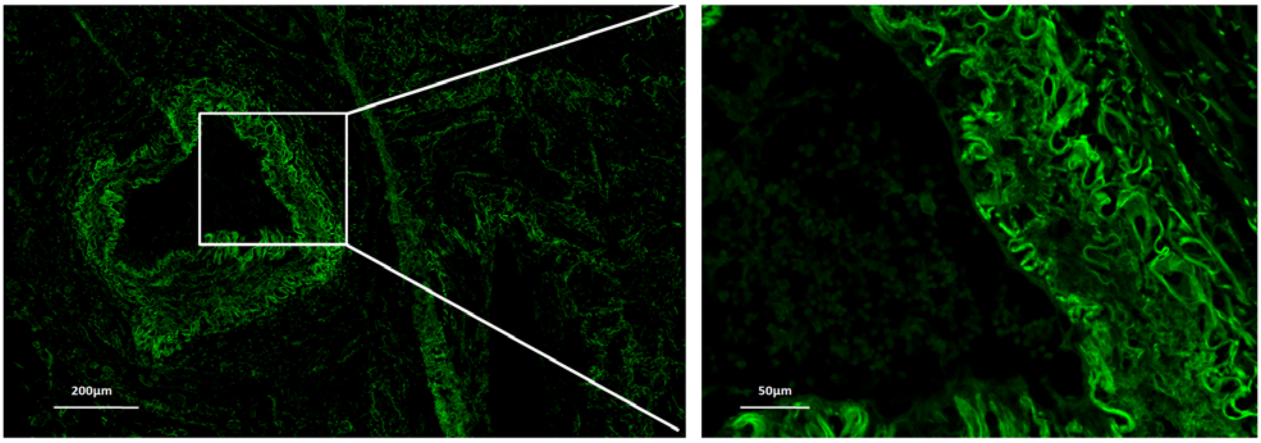

Figure S5. Binding of aptamer LC-224 to arteries and connective tissues: elastic, reticular and collagen fibers from patients with lung adenocarcinoma. Scanning the section (20X).

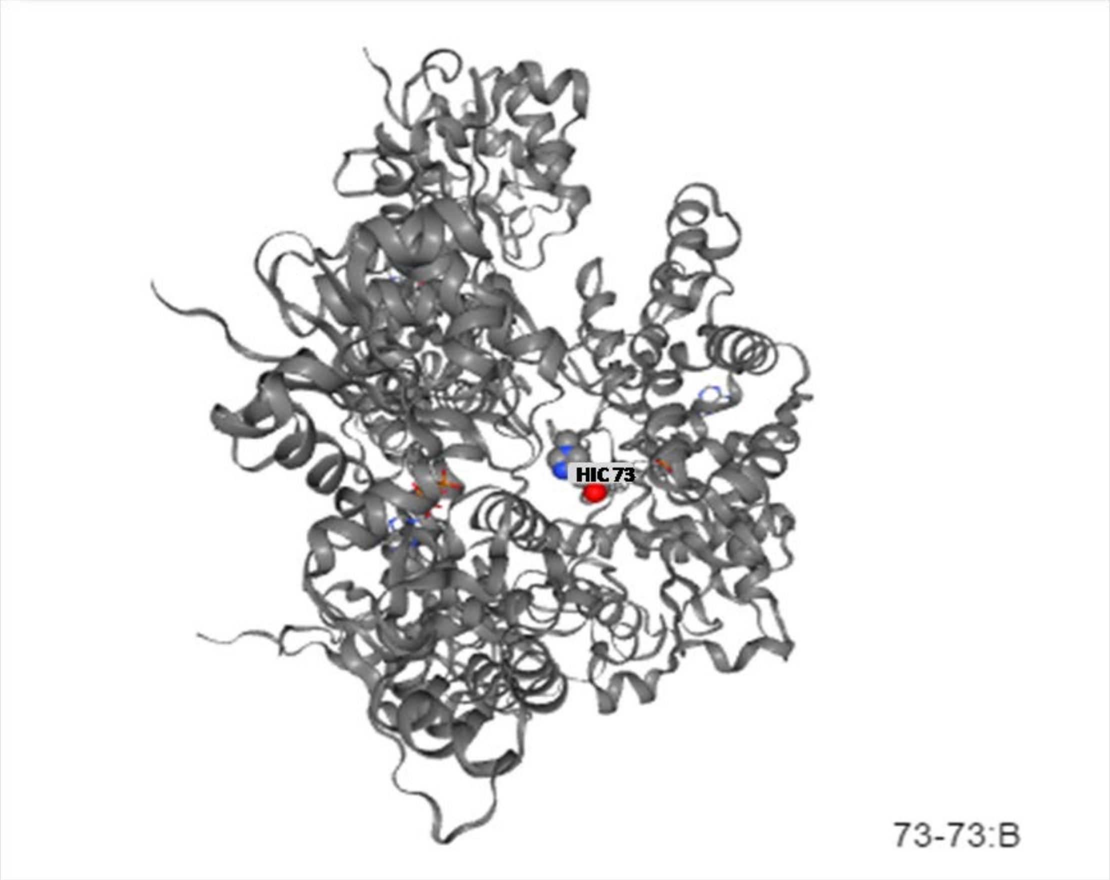

Figure S6. Methylation of 73 histidine in actin, cytoplasmic 1, protein feature view of PDB entries mapped to a UniProtKB sequence. Source:www.rcsb.org/pdb/protein/P60709

A

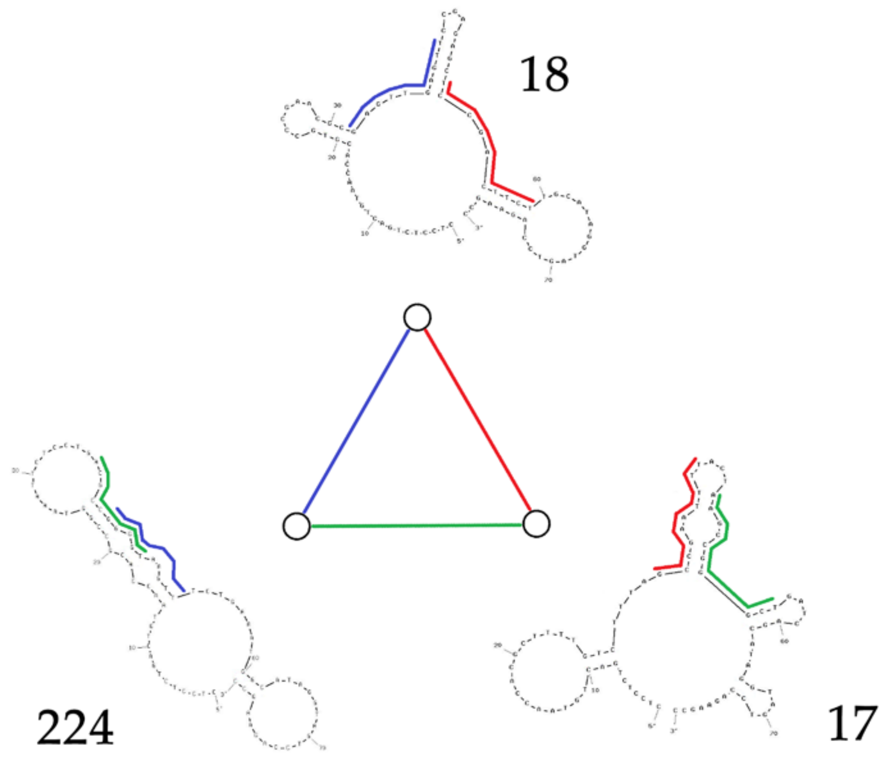

B

17 CTCCTCTGACTGTAACCACGCTTTTGTCTTTAGCCGAATTTTACTAAGCCGGGCTGATCAGCATAGGTAGTCCAGAAGCC

18 CTCCTCTGACTGTAACCACGTGCCCGAACGCGAGTTGAGTTCCGAGAGCTCCGACTTCTTGCATAGGTAGTCCAGAAGCC

224 CTCCTCTGACTGTAACCACGCCGGTAAATTCTCCTGACGCCGGGGTAAGTTTCTGAAATGGCATAGGTAGTCCAGAAGCC

Figure S7. Similar regions for each pair of the aptamers. 10-nucleotide regionsthat differ in the smallest possible number of symbols (from 3 to 2 difference) are shown in primary structure (A) and in the intended secondary structure (B). Each color corresponds to a similar region in a pair: LC-17 and LC-18 – red line; LC-17 and LC-224 – green line; LC-224 and LC-18 – dark blue line.The three aptamers were compared with each other in pairs and we observed thataptamerpairs have similar 10-nucleotide regions. These are shown in primary aptamer structures (B) and in the intended secondary structures (A). Each aptamer has a similar region with other aptamers; aptamer LC-17 has similar regions with LC-18 and LC-224. Despite some similarities in the primary structures, the secondary structures of closely related regions are different.

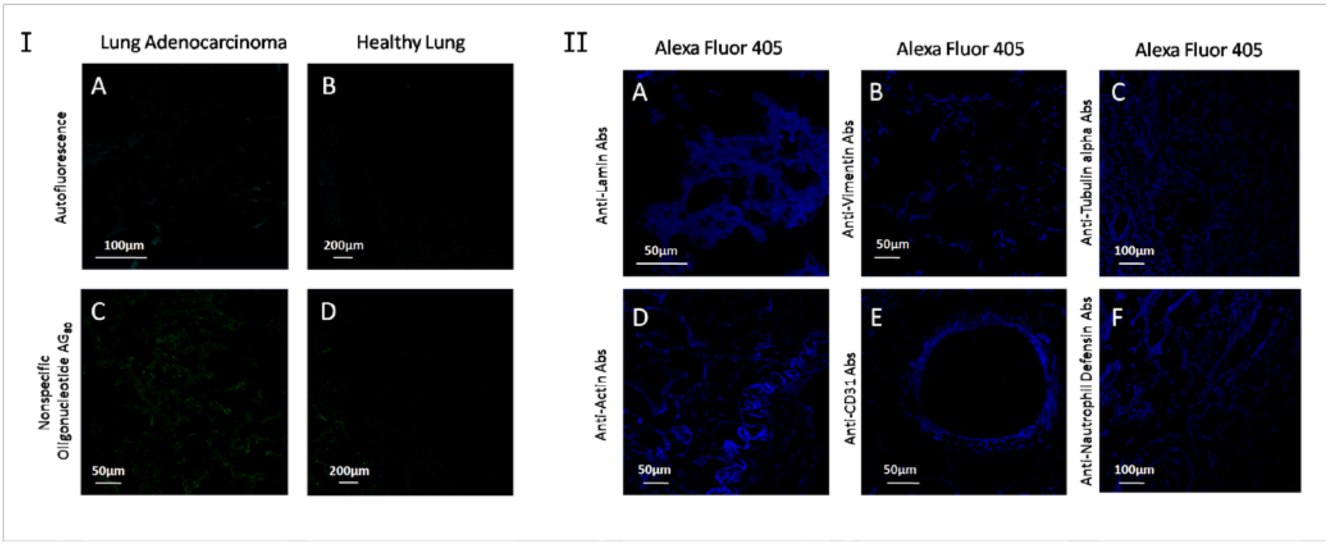

Figure S8. Negative (I) and positive (II) controls for aptahistochemical staining.(I)Autofluorescence of the tissuesections of lung adenocarcinoma (A) and healthy lung (B); binding of non-specific oligonucleotide $(AG)_{40}$to (A) lung adenocarcinoma, (B) healthy lungtissuesections. (II) Positive controls staining of lung adenocarcinoma with antibodies: anti-lamin (A), anti-vimentin (B), anti-tubulin alpha (C), anti-actin (D), anti-CD31 (E), anti-neutrophil defensin (F).
